# Response of Multiple Tissues to Drought Revealed by a Weighted Gene Co-Expression Network Analysis in Foxtail Millet [*Setaria italica* (L.) P. Beauv.]

**DOI:** 10.3389/fpls.2021.746166

**Published:** 2022-01-12

**Authors:** Renliang Zhang, Hui Zhi, Yuhui Li, Erhu Guo, Guojun Feng, Sha Tang, Weixia Guo, Linlin Zhang, Guanqing Jia, Xianmin Diao

**Affiliations:** ^1^Institute of Crop Science, Chinese Academy of Agricultural Sciences, Beijing, China; ^2^Research Institute of Millet, Shanxi Academy of Agricultural Sciences, Taiyuan, China; ^3^Research Institute of Grain Crop, Xinjiang Academy of Agricultural Sciences, Urumqi, China

**Keywords:** *Setaria italica*, WGCNA, drought, multi-tissue, jointing stage, RNA-Seq

## Abstract

Characterization of drought-tolerance mechanisms during the jointing stage in foxtail millet under water-limited conditions is essential for improving the grain yield of this C_4_ crop species. In this trial, two drought-tolerant and two drought-sensitive cultivars were examined using transcriptomic dissections of three tissues (root, stem, and leaf) under naturally occurring water-limited conditions. We detected a total of 32,170 expressed genes and characterized 13,552 differentially expressed genes (DEGs) correlated with drought treatment. The majority of DEGs were identified in the root tissue, followed by leaf and stem tissues, and the number of DEGs identified in the stems of drought-sensitive cultivars was about two times higher than the drought-tolerant ones. A total of 127 differentially expressed transcription factors (DETFs) with different drought-responsive patterns were identified between drought-tolerant and drought-sensitive genotypes (including *MYB, b-ZIP, ERF*, and *WRKY*). Furthermore, a total of 34 modules were constructed for all expressed genes using a weighted gene co-expression network analysis (WGCNA), and seven modules were closely related to the drought treatment. A total of 1,343 hub genes (including *RAB18, LEA14*, and *RD22*) were detected in the drought-related module, and cell cycle and DNA replication-related transcriptional pathways were identified as vital regulators of drought tolerance in foxtail millet. The results of this study provide a comprehensive overview of how *Setaria italica* copes with drought-inflicted environments during the jointing stage through transcriptional regulating strategies in different organs and lays a foundation for the improvement of drought-tolerant cereal cultivars through genomic editing approaches in the future.

## Introduction

Drought is the most costly and deadly environmental phenomenon across the world (Ault, 2020). Agricultural irrigation uses over 70% of the earth's available fresh water each year. This level of agricultural production currently provides sufficient grain food for the increasing human population. Water demand will be a great challenge for the sustainable development of human societies under future water-limited environments (Gong et al., 2020; Gupta et al., 2020). During the history of genetic evolution, plants have established complex regulatory modes of morphological, developmental, physiological, and transcriptional strategies for responding to and then avoiding damages from abiotic environmental stress (Zhu, 2016). Understanding the molecular basis of stress tolerance in crop species is essential for creating crop varieties with higher productivity under unfavorable conditions (Fang and Xiong, 2015).

To clarify vital regulators contributing to drought tolerance in plants, the study of critical factors and regulatory networks involved in plant drought responses has been a research focus in recent decades. The phytohormone abscisic acid (ABA) plays a central role in regulating drought tolerance responses in plants. The 9-cis-epoxycarotenoid dioxygenase enzymes (NCEDs) are crucial catalyzing enzymes of ABA synthesis, and overexpression of NCEDs leads to elevated accumulation of endogenous ABA and enhanced drought tolerance in plants (Thompson et al., 2007). Proline is one of the most vital osmotic adjustment amino acids contributing to drought tolerance of plant species under water-limited conditions (Blum, 2017). The P5CS enzyme regulates the biosynthesis of proline, which could determine the accumulation of proline in plant cells under drought and salt-stressed environments (Funck et al., 2020). Many transcription factor families, such as *WRKY, bZIP, MYB, AP2/EREBP, NAC*, and *HSF*, are essential regulators of drought response in plants (Fang and Xiong, 2015). For example, *ABFs* and *AREBs* are well-known *bZIP* family transcription factors with ABA-dependent regulatory functions; triple mutations of *areb1/areb2/abf3* showed less ABA sensitivity and reduced drought tolerant abilities of plant individuals (Yoshida et al., 2010). Recently, H_2_S, small peptide, and small ncRNA have been identified as vital molecules for long-distance signal transductions of drought-stress conditions in plants (Gong et al., 2020; Zhang et al., 2021), and the formation of various ribonucleic protein complexes is known to be involved in drought-responding courses (Gong et al., 2020). Until now, most known drought-tolerant regulators have not been used in breeding programs because they caused a severe reduction in grain yield under normal conditions.

C_4_ cereal crops are essential food and forage sources for meeting future demands of human societies due to their higher productivity and tolerant ability under abiotic stress conditions (Peng and Zhang, 2021). Foxtail millet (*Setaria italica*) is an ancient crop species cultivated across dry areas of the globe. The species is considered a model crop plant for deciphering plant drought-tolerance mechanisms, owing to the small size of the diploid genome (≈420 Mb) (Bennetzen et al., 2012; Zhang et al., 2012), rapid propagation under indoor conditions, a higher level of genomic diversity (Jia et al., 2013), and the feasible outbreak of genome-modified approach constructed using the accession of *xiaomi* in foxtail millet (Diao et al., 2014; Yang et al., 2020). Many genome-wide transcriptomic studies clarify the genetic basis of drought tolerance in *S. italica*. Zhang et al. found 37 differentially expressed ESTs in the roots and shoots of foxtail millet by constructing a subtractive library under drought conditions (Zhang et al., 2007). Qi et al. identified 2,824 drought-induced genes and 17 lincRNAs in foxtail millet under PEG treatment using the deep sequencing approach (Qi et al., 2013). Yadav et al. identified 55 known and 136 novel mRNAs from two drought-tolerant foxtail millet cultivars under drought conditions during the germinating stage (Yadav et al., 2016). Tang et al. characterized transcriptomic changes between drought-tolerant and drought-sensitive cultivars during the seedling stage, and at least 20 candidate genes overlap with drought tolerance-related quantitative trait locus (QTLs) (Qie et al., 2014) in foxtail millet (Tang et al., 2017).

Previous studies have analyzed the drought-tolerant basis of foxtail millet at an early developmental stage, and most investigations have employed PEGs to simulate osmotic stress conditions, which is different from gradually occurring natural drought conditions in the field. No studies occur during the jointing stage, an essential developmental stage in which the plants demand a high-water supply. Our study used RNA-seq under both normal and drought conditions and aimed to reveal mechanisms of drought tolerance in multiple tissues, including roots, stems, and leaves at the jointing stage in two drought-tolerant cultivars (Ci328 and Ci409) and two drought-sensitive cultivars (Ci134 and Ci603) of foxtail millet (Supplementary Figure S1). The results of this study will provide a comprehensive overview of how *S. italica* adjusts different organs to cope with drought conditions during the jointing stage. We used transcriptional regulating strategies to complete this research and to provide a model to improve drought-tolerant cereal cultivars through genomic editing approaches in the future.

## Materials and Methods

### Plant Materials and Sampling

**Figure 1:**
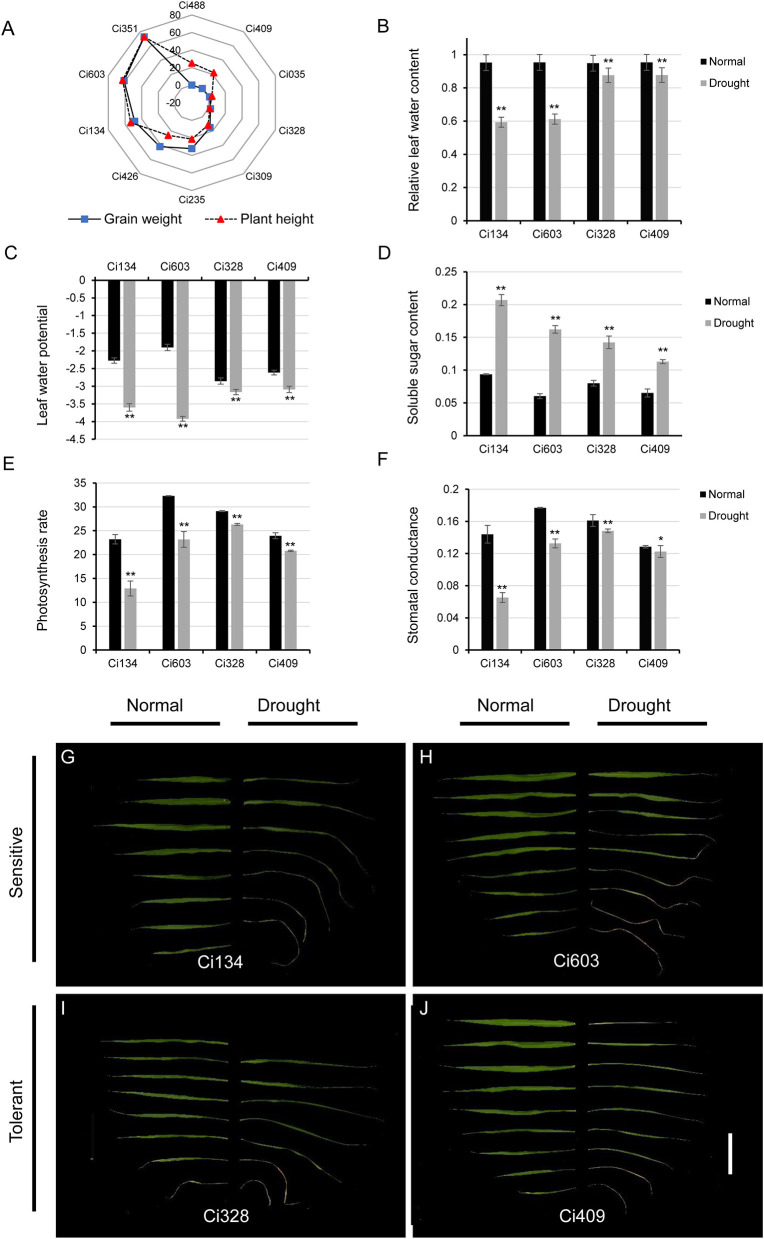
Responses of drought-tolerant and sensitive accessions toward water-limited conditions at the jointing stage. **(A)** A decrease in grain weight and plant height in 10 diverse accessions under water-limited conditions during the jointing stage; **(B–F)** leaf-relative water contents **(B)**, leaf water potential **(C)**, soluble sugar content **(D)**, photosynthetic rate **(E)**, and stomatal conductance **(F)** of drought-sensitive and drought-tolerant cultivars under both normal and drought-stressed conditions; **(G–J)** morphological phenotypes of spreading leaves after drought treatments in drought-sensitive and drought-tolerant accessions. Ci134 **(G)** and Ci603 **(H)** are drought-sensitive genotypes, while Ci328 **(I)** and Ci409 **(J)** are drought tolerant. Bar = 10 cm. * represents *p* < 0.05 determined by Student's *t-*test, ** represents *p* < 0.01 determined by Student's *t*-test.

We selected 10 *S. italica* accessions from a previous study (Jia et al., 2013) for field assessment under normal and water-limited conditions during the jointing stage. The 10 accessions and the decrease of phenotypes are introduced in Table 1, Figure 1A, respectively. We selected Ci134 and Ci603 as drought-sensitive genotypes and Ci328 and Ci409 as drought-tolerant genotypes for this trial.

**Table 1:** Information of 10 genotypes used in field experiments.

**ID**	**Name**	**Source location**
Ci035	BoCaiTui	NeiMengGu, China
Ci134	GuZi*GaoLiang	HeiLongJiang, China
Ci235	YangMaoNuo	HuBei, China
Ci309	YuGuYiHao	HeNan, China
Ci328	HongGaiGu	BeiJing, China
Ci351	90357	HeBei, China
Ci409	JiuGu13	JiLin, China
Ci426	JinGu14	ShanXi, China
Ci488	An04-4783	HeNan, China
Ci603	ZhangGu15	HeBei, China

Drought-sensitive accessions (Ci134 and Ci603) and drought-tolerant accessions (Ci328 and Ci409) were planted in pots (diameter, 30 cm; depth, 40 cm) with five uniform individuals selected from 10 seedlings in each pot after 15 days of seed sowing. All of the pots were placed in the greenhouse at the Institute of Crop Sciences, Chinese Academy of Agricultural Sciences (Beijing, China). After 45 days of growth under normal conditions with weekly watering, we selected uniformly developed and healthy individuals for further analysis. For the drought treatment, we randomly selected five pots from each accession to stop watering for 2 weeks, while we continued to water the control individuals every week until 3 days before sampling (Supplementary Figure S2A).

For the RNA-seq, we collected two upper, fully expanded leaves. We also harvested young, elongating stems from the same plant after removing the sheaths and leaves. We collected all of the roots from the same individual (Supplementary Figure S2B). Finally, three individuals were harvested for each accession as biological replicates. All samples were then immersed in liquid nitrogen immediately after collection and stored at −80°C until total RNA extraction.

### Physiological Measurements of Foxtail Millet Accessions

We measured the photosynthesis rate and the stomatal conductance using the Li-6400 platform (LI-COR Inc., USA). We used the WP4-T Dewpoint Potential Meter (ICT International Inc., USA) to measure leaf water potential. The leaf soluble sugar content was measured using the anthrone colorimetry method described in a previous study (Wen et al., 2005).

We sampled fully expanded leaves and weighed (Wf) them to measure relative leaf water content (RLWC). After immersing the leaves in water for 4 h, we removed the surface water (Wt) and weighed the samples. The samples dried in an oven until a constant weight (Wd) was reached. The RLWC was calculated using this formula:


$$\begin{array}{l} RLWC\;=\;\frac{Wf\;-\;Wd}{Wt\;-\;Wd} \end{array}$$

### RNA Extraction and Library Construction

We extracted the total RNA using a TRIZOL kit (Ambion, Thermo Fisher Scientific Inc., USA) according to the protocol described by instructions. The cDNA library was constructed and then sequenced using the Illumina HiSeq 2100 platform with 150-bp paired-end mode (Illumina Inc., USA), with three biological replications for each sample. Raw data obtained by this study are available on the EMBL-EBI database (http://www.ebi.ac.uk/) with accession No. PRJEB43702.

### RNA-Seq Data Analysis Process, DEGs Identification, and qRT-PCR Validation

After removing the adapter and filtering-quality reads, we mapped clean reads to a reference genome for *S. italica* V2.2 (http://phytozome.jgi.doe.gov) using Hisat2 tools (Kim et al., 2015). All transcripts were assembled using StingTie (Pertea et al., 2015). The gene expression level was quantified by FPKM (Fragments Per Kilobase of exon model per million mapped fragments) and the differentially expressed genes (DEGs) were identified using DEseq2 (Love et al., 2014). The screening threshold for DEGs was fold-changes bigger than two, and the false discovery rate was < 0.05.

Identification of DEGs was conducted through two different approaches. First, the drought condition was compared with normal condition to identify drought induced/repressed DEGs, which were then used for detection of genotype/tissue-specific DEGs, differentially expressed transcription factors (DETFs), and k-means cluster analysis. The transcription factors (TF) list was obtained from PlantTFDB (http://planttfdb.gao-lab.org/). Second, drought-tolerant genotypes were compared with sensitive genotypes to find out DEGs between genotypes under the same condition. After elimination of the genotype differences under normal condition, DEGs identified under drought condition were defined as drought-induced DEGs between genotypes (di-DBG).

We randomly selected the DEGs for validation through qRT-PCR, using primers listed in Supplementary Table 15. We selected *Cullin* and *EF1a* as reference genes in stems and leaves and *SDH* and *EF1a* as reference genes in roots. The first strand of cDNA was synthesized using 1 μg of total RNA with a 20-μl reaction mixture and then was diluted to a 200-μl volume. We used 2 μl of cDNA as a template for the qRT-PCR reaction (95°C, 10 min; then 40 cycles of 95°C, 10 s; 60°C, 60 s). Four replications were conducted for each gene and calculated the relative expression levels using the 2^−ΔΔ*CT*^ method.

### Functional Enrichment Analysis

We conducted functional annotation of transcripts using Gene Ontology (GO, https://geneontology.org) and the Kyoto Encyclopedia of Genes and Genomes (KEGG, https://www.genome.jp/kegg) database. Gene sets functional enrichment analysis was performed with clusterProfiler (Yu et al., 2012), and enrichment results were visualized using ggplot2 (Wickham, 2016).

### K-Means Clustering and Weighted Gene Co-Expression Network Construction

A total of 9,237 DEGs detected in roots, 4,276 DEGs detected in stems, and 6,923 DEGs detected in leaves were used as inputs for K-means cluster analysis, respectively, after being conversed by log_10_ (FPKM + 1), and 6 clusters were finally generated from each DEG set.

We filtered all gene expression data by FPKM > 0.1 in at least eight samples. After normalizing the data by log_2_ (FPKM + 1), the weighted gene co-expression network was constructed using the WGCNA package (Langfelder and Horvath, 2012) with the default parameters except that power = 5, maxBlockSize = 5,000. We exported and visualized the network using Cytoscape software (version 3.7.2, https://cytoscape.org/).

## Results and Discussion

### Identification of Two Drought-Tolerant and Two Drought-Sensitive *S. italica* Accessions Through Morphological and Physiological Analysis

We used the foxtail millet core collections reported in our previous study (Jia et al., 2013) to select drought-sensitive and drought-tolerant accessions. Many elite cultivars of *S. italica* exhibit exceptional drought tolerance abilities under water-limited conditions and rarely decrease grain yield under osmotic conditions, while drought-sensitive accessions incur a heavy grain yield loss for adjusting similar water-deficient conditions. In this trial, we screened out 10 accessions according to the grain-weight decreased ratio (1 – drought/control ×100%), and then we evaluated them in field experiments under normal and drought conditions (Figure 1A). To our surprise, many drought-tolerant genotypes of *S. italica* that we identified at the jointing stage were not consistent with those identified at the seedling stage. For instance, Ci488 is most vulnerable, and Ci309 is generally tolerant to drought conditions at the seedling stage, according to a previous study (Tang et al., 2017), but the grain weight and growth of Ci488 (Figure 1A) were only slightly affected by drought at the jointing stage in the field. This observation indicates that the drought-tolerant abilities of many foxtail millet varieties might not remain stable during the whole development period. Similar results were also characterized in other grass species like sorghum (Varoquaux et al., 2019). Compared with normal conditions, grain weight and plant height of Ci309 and Ci328 were decreased by about 6% under drought environments. Grain weight, and panicle weight of Ci409 was stable, and plant height of Ci409 was decreased by 22% under osmotic conditions compared with the control. Grain weight, panicle weight, and plant height also decreased in Ci134 (≈50%), Ci603 (≈60%), and Ci351 (≈70%) under drought conditions, respectively (Figure 1A). We also evaluated heading dates of relevant accessions (Ci235 and Ci426 were much longer; Ci035 was shorter than others. Data not shown) for accurate determination of developmental stages for drought treatment and sample harvesting. Finally, we selected Ci134 and Ci603 as drought-sensitive genotypes. We selected Ci328 and Ci409 as drought-tolerant genotypes for further RNA-seq analysis.

After 2 weeks of drought treatment, when the soil-water content fell below 15% (Table 2), we sampled from the drought-tolerant genotypes (Ci328 and Ci409) and the drought-sensitive genotypes (Ci134 and Ci603) for RNA-seq. Relative leaf water content (RLWC) of drought-sensitive and drought-tolerant genotypes was comparable (≈95%) under normal conditions, while we observed a difference in the RLWCs between drought-sensitive and drought-tolerant plants after 2 weeks of drought treatment. The RLWCs of two drought-sensitive genotypes decreased by ≈35% (35.8% for Ci134 and 34.1% for Ci603), but drought-tolerant genotypes only decreased by ≈7.5% (7.3% for Ci328 and 7.6% for Ci409) under osmotic-stress conditions (Figure 1B). Leaf water potential (LWP) of drought-tolerant genotypes was higher than drought-sensitive genotypes under drought conditions, and the change of LWPs in tolerant genotypes toward drought treatment was smaller than that of drought-sensitive genotypes (Figure 1C). Stabilities of soluble sugar content in leaves of drought-tolerant genotypes were also higher than drought-sensitive genotypes under drought treatments, implying osmatic adjustments are an essential propagation strategy for optimizing drought-tolerance of foxtail millet under water-limited conditions (Figure 1D). In addition, the photosynthesis rate (Figure 1E) and the stomatal conductance (Figure 1F) of all four accessions under drought treatments were significantly lower than the controls, with tolerant genotypes more stable than sensitive genotypes.

**Table 2:** Water content of soil for treatment of four accessions.

	**Normal**	**Drought**
Ci328	24.6 ± 0.8%	11.2 ± 0.7%
Ci409	25.3 ± 0.6%	13.8 ± 0.4%
Ci134	26.3 ± 0.9%	14.3 ± 0.6%
Ci603	25.7 ± 1.1%	13.4 ± 0.8%

Figures 1G–J illustrate the morphological changes of leaves in sampled accessions after drought treatments. Fully expanded leaves of drought-sensitive genotypes (Ci134 and Ci603) bleached earlier and more severely than drought-tolerant genotypes under similar drought conditions. Notably, fully expanded leaves of the Ci134 genotype died due to dehydration, indicating Ci134 is sensitive to osmotic stress conditions. Based on these findings, we confirmed that Ci134 and Ci603 are drought-sensitive accessions, while Ci328 and Ci409 are drought-tolerant accessions at jointing developmental stages of foxtail millet.

### Differentially Expressed Genes Identified in Stems Might Contribute to Drought Tolerance

**Figure 2:**
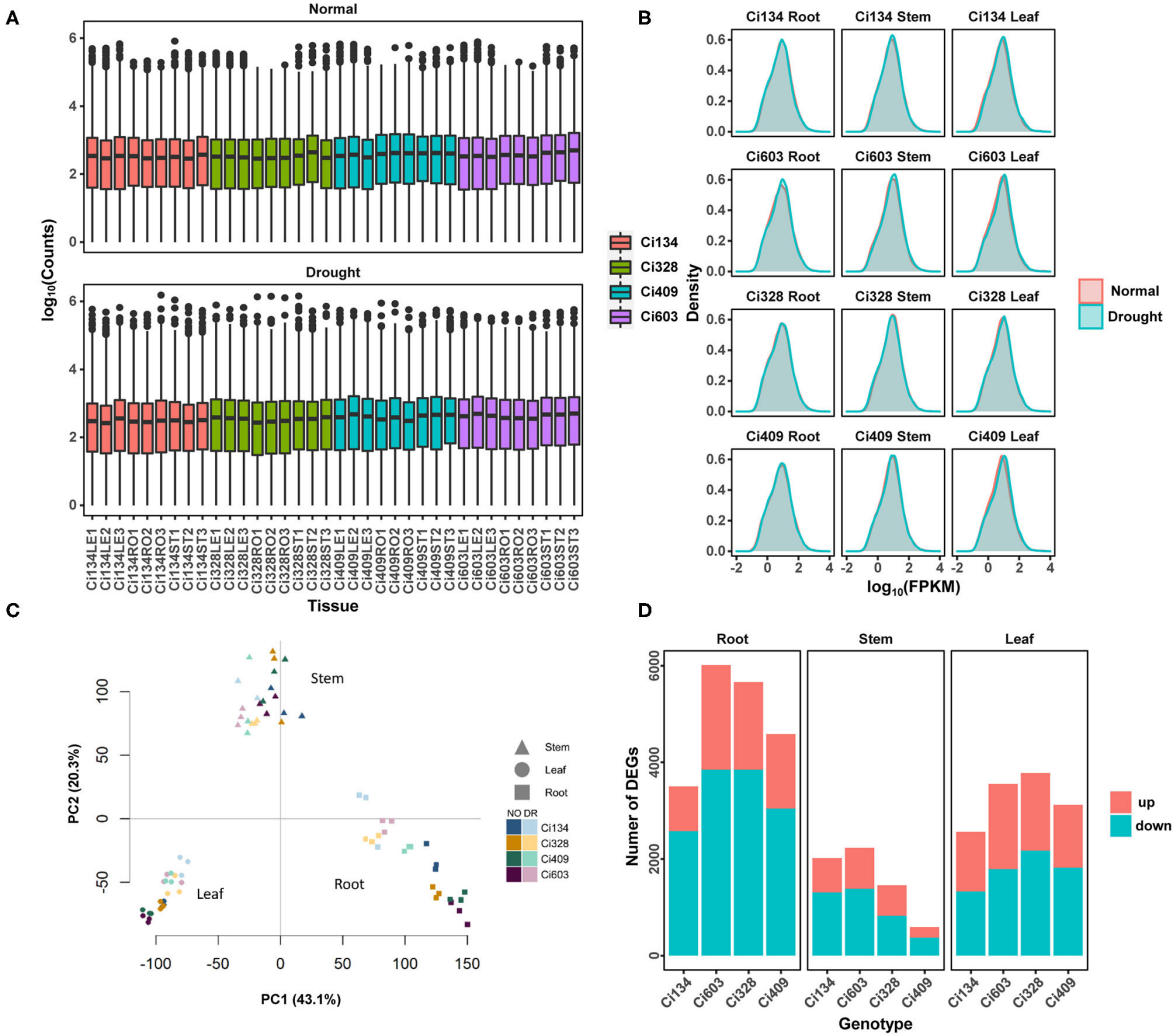
A summary of differentially expressed genes detected in this trial. **(A)** Distributions of log_10_ (counts); **(B)** density of log_10_ (FPKM); **(C)** PCA analysis of each RNA-seq sample. (**D**) Number of DEGs responding to drought treatment in each sample. Drought-tolerant genotypes are Ci328 and Ci409; drought-sensitive genotypes are Ci134 and Ci603. NO represents the normal condition, and DR represents drought treatments.

To achieve a better understanding of drought-tolerant mechanisms of *S. italica* during the jointing stage, we conducted RNA-seq for roots, stems, and leaves of two drought-sensitive (Ci134 and Ci603) and two drought-tolerant genotypes (Ci328 and Ci409) under normal and drought conditions (Supplementary Figure S2). We constructed 72 pair-end libraries for transcriptomic sequencing and generated over 222.24 GB of raw reads. We obtained 1,682,873,236 clean reads and successfully mapped 89.95% of the reads to a reference genome of foxtail millet (*Sitalica_312_v2.2*, https://phytozome.jgi.doe.gov/) (Supplementary Table S1). The number of reads mapped to annotated genes ranged from 1 to ≈1.5 million, with a median of 352 (Figure 2A). We detected a total of 32,170 expressed genes in all samples (Supplementary Table S2), and the log_10_(FPKM) value for all samples was a spread tally with a normal distribution (Figure 2B). We performed principal component analysis using expressed genes shared by all 72 samples (Figure 2C). All samples were divided into three main groups: roots, stems, and leaves. Additionally, each group was then separated into normal and drought treatment subgroups. All three biological replicates were mostly closed based on principal component analysis.

In this trial, we screened out expressed genes with more than 2-fold change (FC) and with a false discovery rate (FDR) of less than 0.05 as differentially expressed genes (DEGs). We separately analyzed three tissues of two drought-tolerant (Ci328 and Ci409) and two drought-sensitive genotypes (Ci134 and Ci603) under both drought and normal conditions at the jointing stage. A total of 13,552 DEGs correlated with drought tolerance among twelve comparison groups were identified (Table 3, Supplementary Table S3). We found the most DEGs in roots, followed by leaves, and then stems of all four genotypes. Overall, the DEGs detected in roots (9,237 DEGs, including 3,497 in Ci134, 6,007 in Ci603, 5,656 in Ci328, and 4,583 in Ci409) were 33% higher than in leaves (6,923 DEGs, including 2,557 in Ci134, 3,549 in Ci603, 3,776 in Ci328, and 3,117 in Ci409) and more than 2-fold higher than in stems (4,276 DEGs, including 2,013 in Ci134, 2,229 in Ci603, 1,454 in Ci328, and 587 in Ci409) (Table 3, Figure 2D). These observations suggest that the roots are more sensitive to drought conditions, consistent with observations reported in rice (Wang D. et al., 2011). Moreover, the number of DEGs in the stems of two drought-sensitive genotypes (2,013 DEGs in Ci134 and 2,229 DEGs in Ci603, respectively) was higher than that of two drought-tolerant genotypes (1,454 DEGs in Ci328 and 587 DEGs in Ci409), suggesting stems might play an important role in determining drought tolerance in foxtail millet (Table 3).

**Table 3:** Number of DEGs detected in all four accessions.

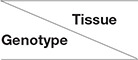		**Root**	**Stem**	**Leaf**	**Total**
Ci134		3,497	2,013	2,557	6,481
Ci603		6,007	2,229	3,549	9,184
Ci328		5,656	1,454	3,776	8,497
Ci409		4,583	587	3,117	6,953
Total		9,237	4,276	6,923	13,552

### Different Biological Pathways Were Influenced by Drought in Three Tissues

To clarify the biological functions of the DEGs responses to drought in foxtail millet, we performed KEGG and GO enrichment analysis for each DEGs set. The functional enrichment analysis of DEGs detected in roots revealed osmotic adjustment and phytohormone-related pathways, playing important roles in drought-tolerant mechanisms of *S. italica*. KEGG enrichment analysis revealed “starch and sucrose metabolism,” and “galactose metabolism” pathways were enriched in upregulated DEGs, and “phenylpropanoid and diterpenoid biosynthesis” pathways were enriched in downregulated DEGs identified in roots of foxtail millet. As a whole, “glycolysis and glutathione metabolism” pathways were enriched in all DEGs detected in roots of all four accessions (Supplementary Figure S3A, Supplementary Table S4). Moreover, GO enrichment analysis also revealed that regulatory genes participated in responding pathways of heat, water, and growth hormones were obviously affected by drought stress (Supplementary Figure S3B, Supplementary Table S5). Interestingly, two ABA pathway-related genes of *ABF4* (*Seita.1G331600*) and *ABA2* (*Seita.9G041900*) were identified as downregulated DEGs in drought-sensitive genotypes but upregulated DEGs in drought-tolerant genotypes.

Functional categories of the DEGs identified in the stems of drought-sensitive (Ci134 and Ci603), and drought-tolerant (Ci328 and Ci409) genotypes revealed that young stem elongation was affected in drought-sensitive genotypes, and heat shock proteins (HSPs) might contribute to the growth of young stems of foxtail millet under drought conditions. Regulatory genes involved in plant cell organizations were primarily enriched in the DEGs set of drought-sensitive genotypes, and the number of downregulated DEGs of this pathway was higher in drought-sensitive genotypes than those that are drought tolerant. This observation implies that stem elongation of foxtail millet under drought conditions was seriously affected, which coincides with the plant height of both drought-sensitive and drought-tolerant accessions detected in this trial under normal and drought conditions (Figure 1A). KEGG enrichment analysis revealed “plant hormone signal transduction” and “amino sugar and nucleotide sugar metabolism” pathways were enriched in upregulated DEGs detected in the stems of drought-tolerant genotypes (Supplementary Figure S4A, Supplementary Table S4). Interestingly, heat and reactive oxygen species response GO terms were only enriched in drought-tolerant accessions (Supplementary Figure S4B, Supplementary Table S5), and five HSPs (*Seita.3G175700, Seita.5G131400, Seita.9G470600, Seita.9G488400*, and *Seita.2G241600*) were identified as upregulated DEGs in drought-tolerant genotypes under stressed conditions, while no significant change was detected in drought-sensitive genotypes. Therefore, we speculate that drought-sensitive genotypes might reduce chaperone synthesis, which could support elongation of stems under drought conditions through a transcription regulatory mode.

Functional enrichment analysis of drought-response DEGs in leaves of all genotypes revealed a clear signature that photosynthesis-related genes were downregulated, but plant hormone transduction-related genes were upregulated under drought conditions (Supplementary Tables S4, S5). Moreover, the “starch and sucrose metabolism” pathway was enriched for downregulated DEGs in sensitive genotypes. In contrast, the “glycerol phospholipid metabolism” pathway was enriched for upregulated DEGs in drought-tolerant genotypes (Supplementary Figure S5), suggesting photosynthetic characters of functional leaves were seriously affected under water-limited conditions.

### The Majority of DEGs Were Identified in a Tissue or Genotype-Specific Manner

**Figure 3:**
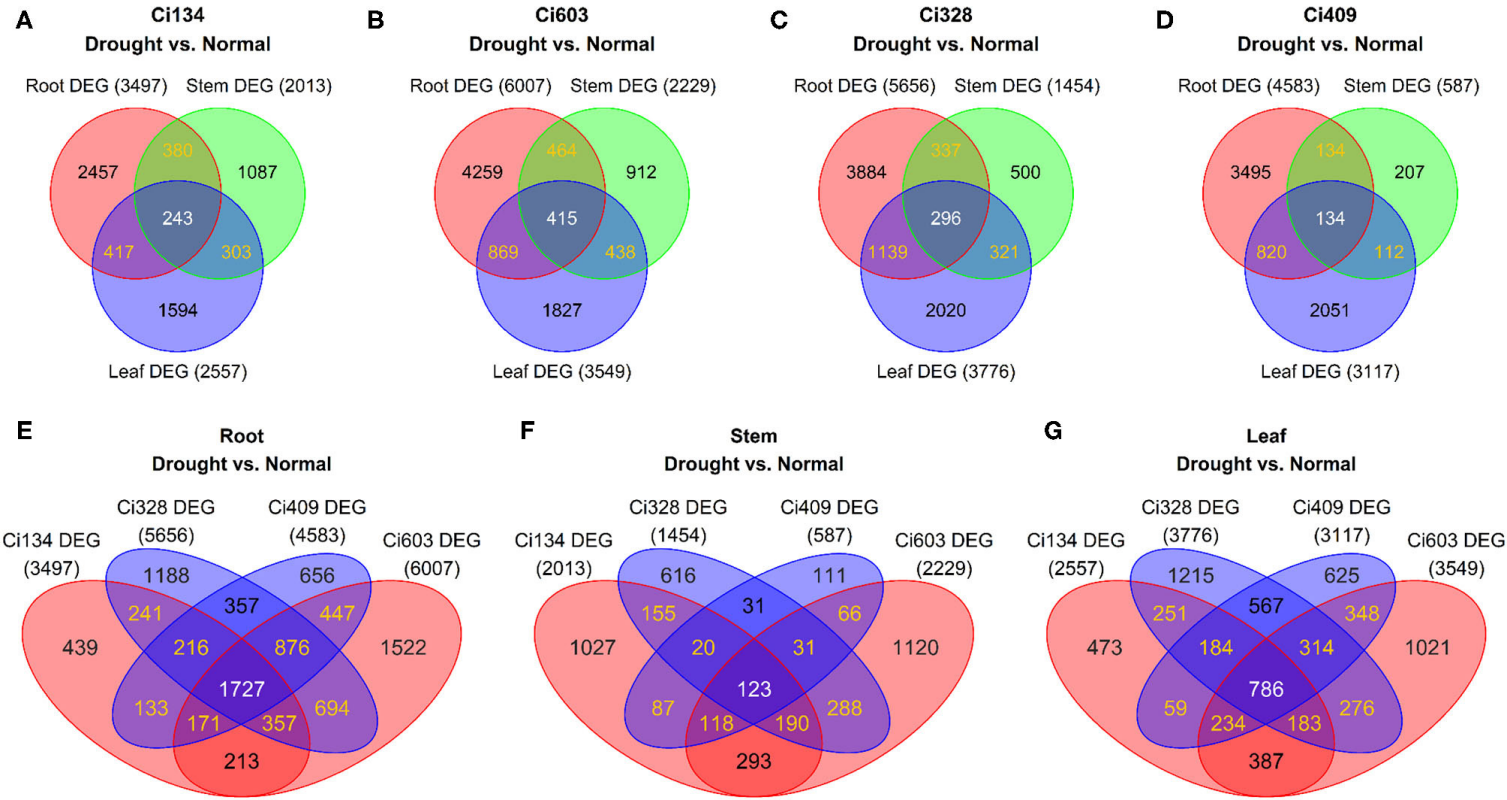
Comparisons of DEGs from three tissues of four foxtail millet accessions. Venn diagrams of shared DEGs among different genotypes (**A**: Ci134; **B**: Ci603; **C**: Ci328; **D**: Ci409) and different tissues (**E**: root; **F**: stem; and **G**: leaf). Drought-tolerant genotypes: Ci328 and Ci409; drought-sensitive genotypes: Ci134 and Ci603. Numbers in parentheses represent total DEGs.

In this investigation, we detected most DEGs in a tissue-specific manner. In total, only 243, 415, 296, and 134 DEGs out of 6,481, 9,184, 8,497, and 6,953 DEGs were shared in three tissues of Ci134, Ci603, Ci328, and Ci409, respectively (Table 3, Figures 3A–D). There were 551, 1,520, 1,256, and 1,115 upregulated DEGs, 2,011, 3,224, 3,026, and 2,574 downregulated DEGs from roots of Ci134, Ci603, Ci328, and Ci409. Moreover, there were 851, 1,099, 1,010, and 887 induced DEGs, and 893, 1,171, 1,348, and 1,364 repressed DEGs in the leaves of Ci134, Ci603, Ci328, and Ci409 under drought conditions. In stems, there were 386, 350, 223, and 45 upregulated DEGs, and 799, 736, 365, and 188 DEGs were downregulated in Ci134, Ci603, Ci328, and Ci409 under osmotic stress conditions. In total, few of the DEGs were up or downregulated under drought conditions in all three tissues, including 144, 197, 174, and 94 induced DEGs and 88, 130, 78, and 32 repressed DEGs in all four accessions (Ci134, Ci603, Ci328, and Ci409) (Supplementary Figures S6A–D, S7A–D).

We also identified genotype-specific DEGs in this trial. In roots, there were 163 upregulated DEGs and 198 downregulated DEGs only detected in drought-tolerant accessions, while we identified 55 upregulated DEGs and 154 downregulated DEGs in drought-sensitive accessions (Supplementary Figures S6E, S7E). In stems, there were 26 upregulated DEGs and 10 downregulated DEGs only identified in drought-tolerant genotypes, while we only detected 73 upregulated DEGs and 217 downregulated DEGs in drought-sensitive accessions (Supplementary Figures S6F, S7F). In leaves, there were 237 upregulated DEGs and 381 downregulated DEGs only detected in drought-tolerant accessions, while we only identified 194 upregulated DEGs and 253 downregulated DEGs in drought-sensitive genotypes (Supplementary Figures S6G, S7G). The roots of all four genotypes shared a total of 1,727 DEGs (out of 9,237). The leaves of all four accessions shared a total of 786 DEGs (out of 6,923). Furthermore, the stems of all four genotypes shared a total of 123 DEGs (out of 4,276) (Table 3, Figures 3E–G).

To dissect the function of genotype-shared/specified DEGs, KEGG and GO enrichment analysis was then performed (Supplementary Figure S8, Supplementary Table S6), which revealed that water deprivation-related genes were differentially expressed in all four genotypes under drought conditions, and only few of water-deprivation DEGs were shared by different tissues. Moreover, photosynthesis-related genes were affected by drought in leaves of all four accessions, and plant hormone transduction and MAPK-related DEGs were also enriched in leaves and stems.

We detected 35 upregulated and 3 downregulated genes shared by all three tissues of the four accessions under drought treatments (Table 4). Previous studies have confirmed that many functional genes of this DEGs set are correlated with the response of plants to multiple abiotic stress conditions, such as salt, drought, and extreme temperature stresses (Wu et al., 2005; Ali et al., 2013; Nawkar et al., 2017). For instance, 5 LEA proteins, 5 HSPs (HSP70, HSP70T-2, HSA32, and HSP17.6C), 2 PP2Cs (HAI3 and AHG1), and 1 dehydrin family protein (RAB18) were upregulated under drought conditions. Previous study has confirmed that these proteins act as abiotic stress-responding factors (Table 4).

**Table 4:** Induced and repressed DEGs in all three tissues of four accessions.

**GeneID**	**Arabidopsis^a^**	**Gene name**	**Annotation**	**Reference**
**Drought induced DEGs shared in all tissues and accessions**				
*Seita.1G012200*	*AT2G46240*	*BAG6*	BCL-2-associated athanogene 6	Echevarría-Zomeño et al., 2016
*Seita.2G141500*	–	–	–	–
*Seita.2G304700*	*AT5G36100*	–	–	–
*Seita.2G437900*	*AT2G36640*	*ECP63*	Embryonic cell protein 63	Nowak and Gaj, 2016
*Seita.3G139000*	*AT2G29380*	*HAI3*	Highly ABA-induced PP2C gene 3	Yang et al., 2019
*Seita.3G142400*	*AT4G22220*	*ISU1*	SufE/NifU family protein	Leaden et al., 2014
*Seita.3G209800*	*AT5G01300*	–	Phosphatidylethanolamine-binding protein family protein	–
*Seita.3G264300*	*AT1G12920*	*ERF1-2*	Eukaryotic release factor 1-2	Zhou et al., 2010
*Seita.3G357700*	*AT2G03530*	*UPS2*	Ureide permease 2	Schmidt et al., 2004
*Seita.4G159500*	*AT3G22490*	*RAB28*	Seed maturation protein	Borrell et al., 2002
*Seita.4G258900*	*AT4G21320*	*HSA32*	Aldolase-type TIM barrel family protein	Wu et al., 2013
*Seita.5G011800*	*AT1G64110*	*DAA1*	P-loop containing nucleoside triphosphate hydrolases superfamily protein	Ali et al., 2013
*Seita.5G012100*	*AT1G64110*	*DAA1*	P-loop containing nucleoside triphosphate hydrolases superfamily protein	Ali et al., 2013
*Seita.5G093000*	*AT1G53540*	*HSP17.6C*	HSP20-like chaperones superfamily protein	Wu et al., 2020
*Seita.5G298300*	*AT5G11460*	–	Protein of unknown function (DUF581)	–
*Seita.5G346500*	*AT1G60420*	–	DC1 domain-containing protein	–
*Seita.5G422900*	–	–	–	–
*Seita.6G005300*	*AT5G51760*	*AHG1*	Protein phosphatase 2C family protein	Nishimura et al., 2007
*Seita.6G123500*	*AT3G22490*	*RAB28*	Seed maturation protein	Borrell et al., 2002
*Seita.7G121700*	*AT5G65100*	–	Ethylene insensitive 3 family protein	–
*Seita.7G184600*	*AT2G46680*	*HB-7*	homeobox 7	Ré et al., 2014
*Seita.7G320200*	*AT3G10020*	–	–	–
*Seita.8G030400*	*AT3G10020*	–	–	–
*Seita.8G115400*	*AT5G66400*	*RAB18*	Dehydrin family protein	Gosti et al., 1995
*Seita.8G124200*	*AT1G26910*	*RPL10B*	Ribosomal protein L16p/L10e family protein	Falcone Ferreyra et al., 2010
*Seita.9G043000*	*AT4G36730*	*GBF1*	G-box binding factor 1	Smykowski et al., 2016
*Seita.9G056300*	*AT3G22850*	–	Aluminum induced protein with YGL and LRDR motifs	–
*Seita.9G112100*	*AT5G66780*	–	–	–
*Seita.9G134900*	*AT5G62670*	*HA11*	H(+)-ATPase 11	Rodrigues et al., 2014
*Seita.9G410800*	*AT4G02280*	*SUS3*	Sucrose synthase 3	Angeles-Núñez and Tiessen, 2012
*Seita.9G416100*	*AT1G09780*	*IPGAM1*	Phosphoglycerate mutase, 2,3-bisphosphoglycerate-independent	Zhao and Assmann, 2011
*Seita.9G431100*	*AT4G16160*	*ATOEP16-S*	Mitochondrial import inner membrane translocase subunit Tim17/Tim22/Tim23 family protein	Pudelski et al., 2012
*Seita.9G443500*	*AT2G38905*	–	Low temperature and salt responsive protein family	–
*Seita.9G451500*	*AT5G02500*	*HSC70*	Heat shock cognate protein 70-1	Zhao et al., 2021
*Seita.9G488400*	*AT2G32120*	*HSP70T-2*	Heat-shock protein 70T-2	Song et al., 2010
**Drought repressed DEGs shared in all tissues and accessions**				
*Seita.1G354100*	*AT5G34940*	*GUS3*	Glucuronidase 3	Woo et al., 2007
*Seita.3G097400*	*AT4G12910*	*scpl20*	Serine carboxypeptidase-like 20	Cassin-Ross and Hu, 2014
*Seita.9G327100*	*AT1G65680*	*ATEXPB2*	Expansin B2	Han et al., 2015

a *Homological gene ID in Arabidopsis*.

**Figure 4:**
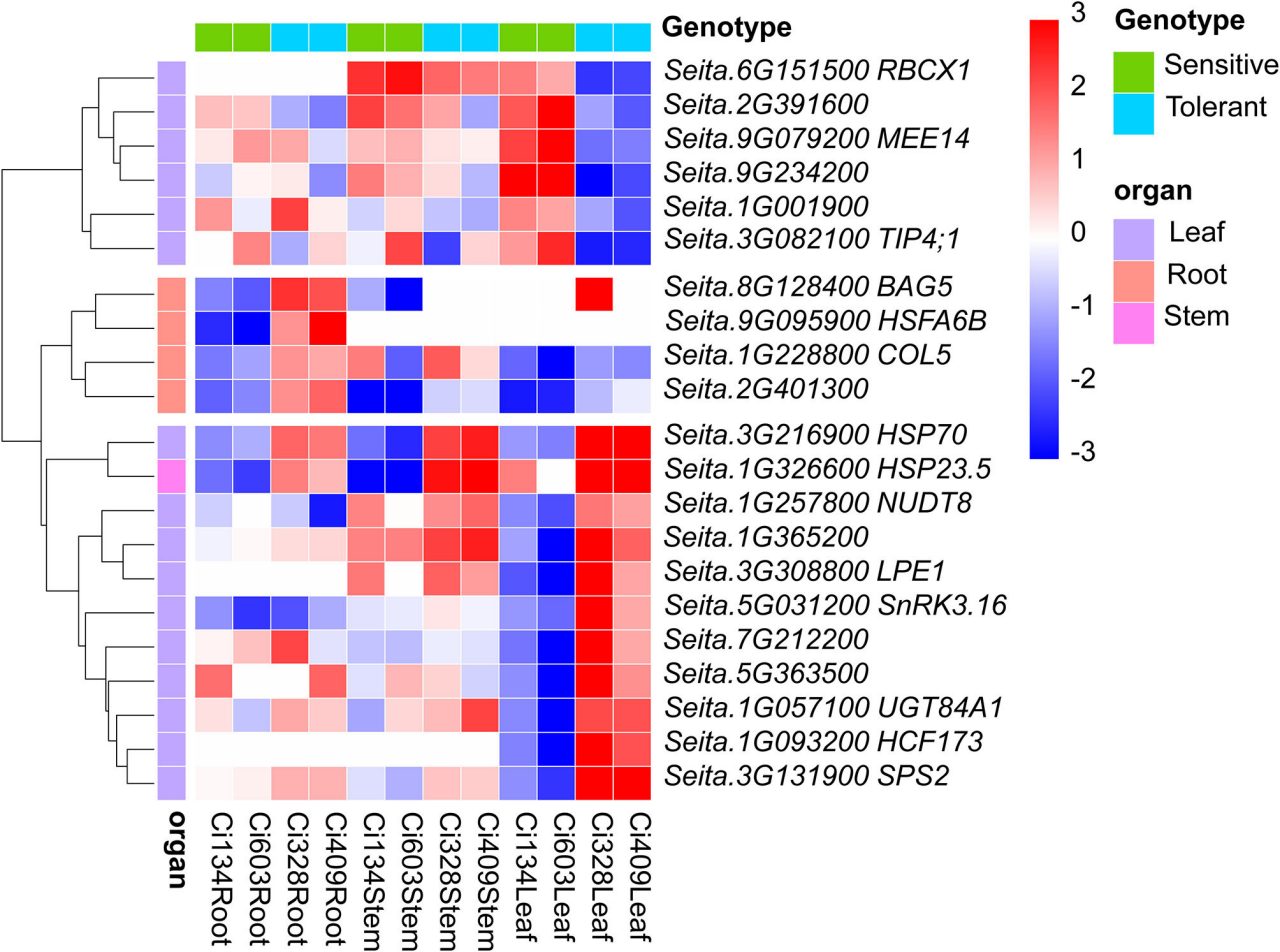
Twenty-one oppositely regulated DEGs were identified in drought-tolerant and drought-sensitive genotypes under water-limited conditions. Cell color represents the expression pattern of relevant DEGs in all three tissues of four accessions. Red represents an upregulated pattern, while blue represents a downregulated pattern. Four, one, and 16 oppositely expressed DEGs responding to drought between drought-sensitive/tolerant accessions that were identified in roots, stems, and leaves, respectively.

Notably, we detected 21 DEGs (including 4, 1, and 16 DEGs identified in roots, stems, and leaves, respectively), responding to drought with the opposite pattern between sensitive and tolerant genotypes in this study (Figure 4, Supplementary Table S7). Two heat shock protein-encoding genes [*Seita.3G216900* and *Seita.1G326600* (*HSP23.5*)] were upregulated in drought-tolerant genotypes but were downregulated in drought-sensitive genotypes under drought conditions. Heat shock protein HSP23.5 has also been confirmed to be induced by salinity and heat conditions in *Arabidopsis* (Sewelam et al., 2019). Moreover, one heat shock transcription factor (*HSFA6B, Seita.9G095900*)-has been characterized, and knock-out of *HSFA6B* in *Arabidopsis* has elevated ABA sensitivity and stress tolerance of transgenic plants (Wang et al., 2020). *Seita.5G031200* (*CIPK1*) encodes a CBL-interacting protein kinase involved in an ABA-dependent regulatory network in the plant (D'Angelo et al., 2006). In addition, *BAG5* (*Seita.8G128400*) is a known leaf-senescence-related regulator in *Arabidopsis* (Fu et al., 2017). We also found *BAG5* was downregulated in the roots of sensitive genotypes and upregulated in tolerant genotypes in this trial, implying that *BAG5* plays a vital role in drought adjustment of the roots in *S. italica*. Moreover, one chloroplast thylakoid-localized RbcX protein (*RBCX1, Seita.6G151500*), a chaperone in the folding of Rubisco, and two pentatricopeptide repeats (PPR like), protein-encoding genes (*LPE1, Seita.3G308800*; *Seita.7G212200*) were also identified as DEGs in the leaves of foxtail millet under drought conditions. These oppositely regulated DEGs between sensitive and tolerant accessions may play key roles in modulations of drought-tolerant abilities of *S. italica* during the jointing stage.

### Transcription Factors Play Essential Roles in the Drought-Tolerance Processes

**Figure 5:**
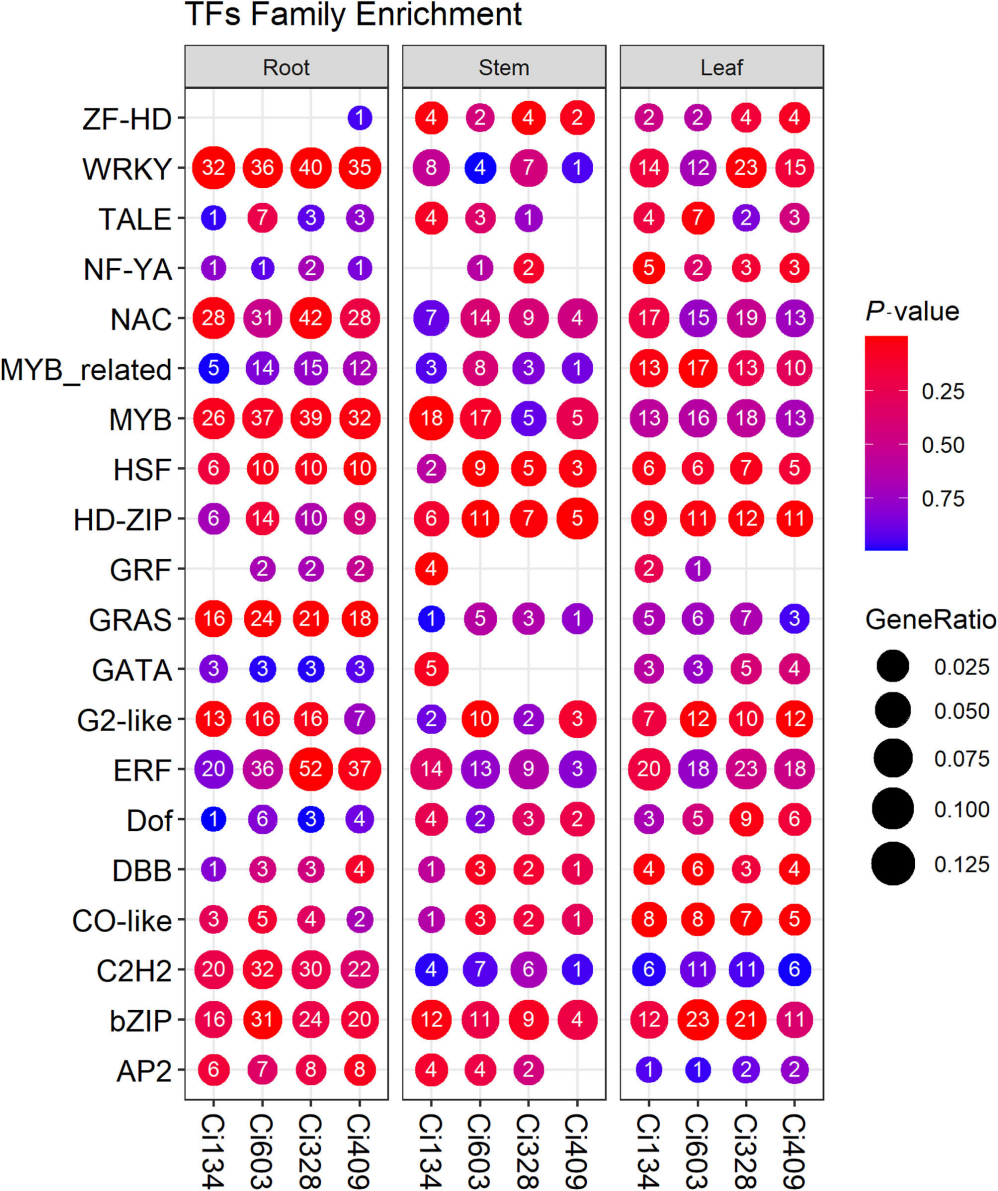
Enrichment of differentially expressed transcription factors (DETFs) identified in three tissues of all four accessions. Each column represents a gene set. The circle size represents the gene ratio, and the circle color represents the *P*-value. The number in the middle of each circle represents the numbers of DETFs annotated in each GO term or KEGG pathway.

Transcription factors play central roles in dealing with abiotic stress conditions through regulating functional genes in plants (Baillo et al., 2019). We identified a total of 915 TFs from the DEGs detected in this trial, including many *WRKY, ERF, MYB, NAC, bHLH, bZIP*, and *HSF* transcription factors that have been confirmed to be important for the tolerance of biotic and abiotic stresses in rice (Todaka et al., 2015). These differentially expressed transcription factors (DETFs) identified in foxtail millet during the jointing stage (Figure 5) were similar to observations reported in previous studies of drought tolerance in foxtail millet at the seedling stage (Tang et al., 2017). We detected 200 DETFs shared by all four accessions (Supplementary Figure S9), including 131 downregulated DETFs and 69 upregulated DETFs in this trial. We identified a total of 35 *ERF*, 35 *WRKY*, 32 *bHLH*, 31 *MYB*, 30 *NAC*, 23 *C2H2*, and 18 *bZIP* DETFs differentially expressed in roots, in at least two genotypes, and 10 *MYBs*, 7 *ERFs*, 6 *HD-ZIPs*, 5 *bHLHs*, and 5 *bZIPs*, and we detected 4 *NAC* family DETFs differentially expressed in stems in more than two accessions, and we identified 18 *ERF*, 15 *MYB*, 13 *bZIP*, 12 *NAC*, and 12 *HD-ZIP* family DETFs differentially expressed in leaves in this trial.

Additionally, we identified 127 DETFs (including 51 in roots, 26 in stems, and 50 in leaves) with different expression patterns in response to drought conditions between drought-tolerant and drought-sensitive accessions (Supplementary Table S8). For instance, *Seita.6G055700* (*LATE ELONGATED HYPOCOTYL*) encodes an *MYB*-like transcription factor, which represses the biosynthesis of ABA in plants (Adams et al., 2018); another *MYB* transcription factor-encoding gene (*Seita.2G199900*) identified as a drought-responding regulator in this study has also been confirmed as a negative regulator of ABA signal transduction; overexpressing of which could enhance salt tolerance of *Arabidopsis* individuals (Cui et al., 2013). Furthermore, in this study, the number of *ERF*-encoded DETFs identified in tolerant genotypes was higher than that of sensitive ones. For instance, an *ERF*-encoding gene (*Seita.9G500100, ERF-IXc5*) has been inferred as a crucial regulator of plant growth and drought tolerance of *Hevea brasiliensis* (Lestari et al., 2018). Moreover, *ERF* family proteins have been considered as crucial regulators responding to environmental stimuli in *Arabidopsis* and rice (Nakano et al., 2006). Notably, two DETFs, including *Seita.1G228800* (*CO*-like family) and *Seita.9G095900* (*HSF* family), were downregulated in the roots of sensitive genotypes but upregulated in tolerant genotypes. Overall, DETFs identified in this trial also suggested that TFs have played essential roles in the drought-tolerant process in foxtail millet during the jointing stage.

### Stress-Related Biological Pathways Are Enriched in DEG Clusters With Similar Expressional Patterns in Three Tissues

**Figure 6:**
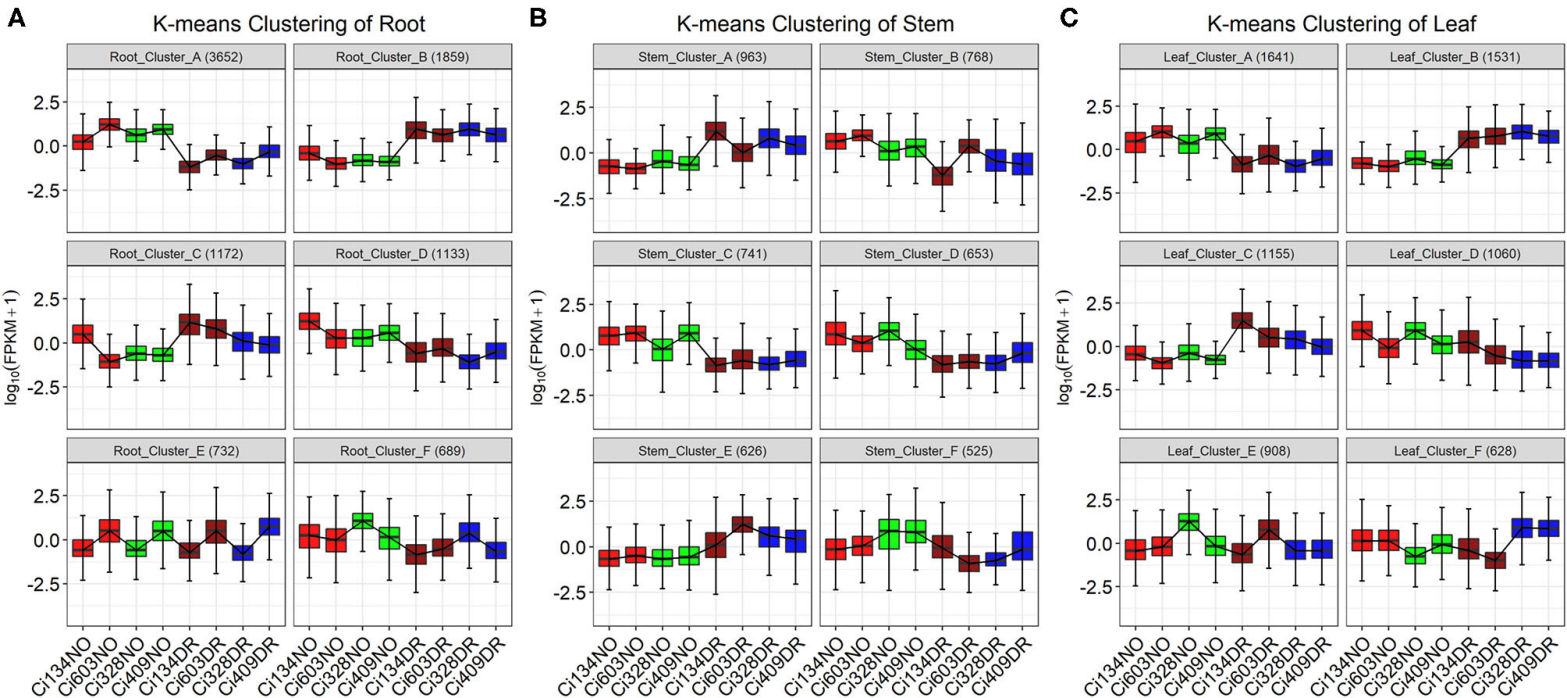
K-means clustering of DEGs identified in three tissues of four foxtail millet accessions. K-means clusters of DEGs in roots **(A)**, stems **(B)**, and leaves **(C)** across all four accessions. NO refers to normal, and DR refers to drought conditions. Numbers in parentheses represent the numbers of DEGs found in each cluster. The y axis represents normalized gene expression. Red and brown refer to normal and drought treatment of drought-sensitive genotypes, respectively. Green and blue refer to normal and drought treatment of drought-tolerant genotypes.

To further dissect biological processes and pathways correlated with drought tolerance in each tissue, a k-means clustering analysis of DEGs detected in leaves, stems, and roots were carried out, respectively, and 6 clusters were generated for each DEG set (Figure 6, Supplementary Table S9). Functional enrichment analysis of each cluster (Supplementary Table S10) revealed that different biological pathways were responsible for drought tolerance in the three different tissues.

In this trial, the biological processes of “water deprivation response” (drought) and “response to heat” (heat) were enriched in many clusters identified in three tissues, which implied that drought and heat are both severe environmental stresses often occurring in the field together. Many clusters, including Cluster B in roots (46 DEGs involved in drought and 33 DEGs involved in heat), Cluster A in stems (30 DEGs involved in drought and 22 DEGs involved in heat), and Cluster B in leaves (37 DEGs involved in drought and 27 DEGs involved in heat), were involved in these two pathways (Figure 6, Supplementary Table S10), which were consistent with upregulated DEGs identified under drought treatments. However, only 11 shared DEGs involved in the “response to water deprivation” pathway and 7 shared DEGs involved in the “response to heat” pathway were identified in all three tissues, implying that different tissues might take different actions to achieve the similar goal of drought tolerance in foxtail millet at the jointing stage.

In leaves, “MAPK signal transduction” and “plant hormone signal transduction” were mainly enriched in Clusters A and B, with DEGs induced or repressed, respectively, by drought treatments. Moreover, “nitrogen metabolism” and “photosynthesis” related pathways were enriched in Cluster D with downregulated DEGs influenced by drought conditions, implying that enhanced signal transduction and decreased efficiency of photosynthesis and energy metabolism in leaves are essential for drought tolerance of foxtail millet under water-limited conditions.

In stems, stress-related pathways, such as “response to water deprivation” and “heat, salt, and hydrogen peroxide,” were enriched in Clusters A and F. Pathways of “secondary cell wall biogenesis” and “xylan biosynthetic process” were also enriched in Cluster F with downregulated DEGs identified under drought conditions, indicating suppression of stem cell growth in foxtail millet under drought conditions during the jointing stage. Furthermore, the “Auxin-activated process” and “secondary cell wall biogenesis” pathways were enriched in Cluster D with downregulated DEGs identified under drought conditions, which was consistent with the fact that plant height decreased under drought conditions through suppressing stem elongation at the jointing stage in foxtail millet. Photosynthesis-related biological pathways, such as “light-harvesting process” and “antenna proteins,” were enriched in Cluster C with downregulated DEGs, and “light reaction” and “carbon fixation” categories were enriched in Cluster E with upregulated DEGs identified under drought conditions, implying that stems were also a photosynthesis-related organ during the jointing stage in foxtail millet.

In roots, “plant-pathogen” categories were enriched in Cluster A with downregulated DEGs under drought conditions, which was consistent with conclusions that the mass of arbuscular mycorrhizal symbiosis with roots decreased under drought conditions in sorghum (Varoquaux et al., 2019). Biological pathways of “plant secondary cell wall biogenesis” and “plant hormone signal transduction” were enriched in Cluster C with DEGs oppositely expressed in the roots and stems, implying that expansion of the root system is essential for water exploration under drought conditions and growth of stems might be restricted by water-deficient environments. Previous research has reported similar findings that the growth of crown roots in *S. italica* and *Maize* is sensitive to drought conditions (Sebastian et al., 2016).

### Regulators Contributing to Genotypic Variations of Drought Tolerance at the Jointing Stage

**Figure 7:**
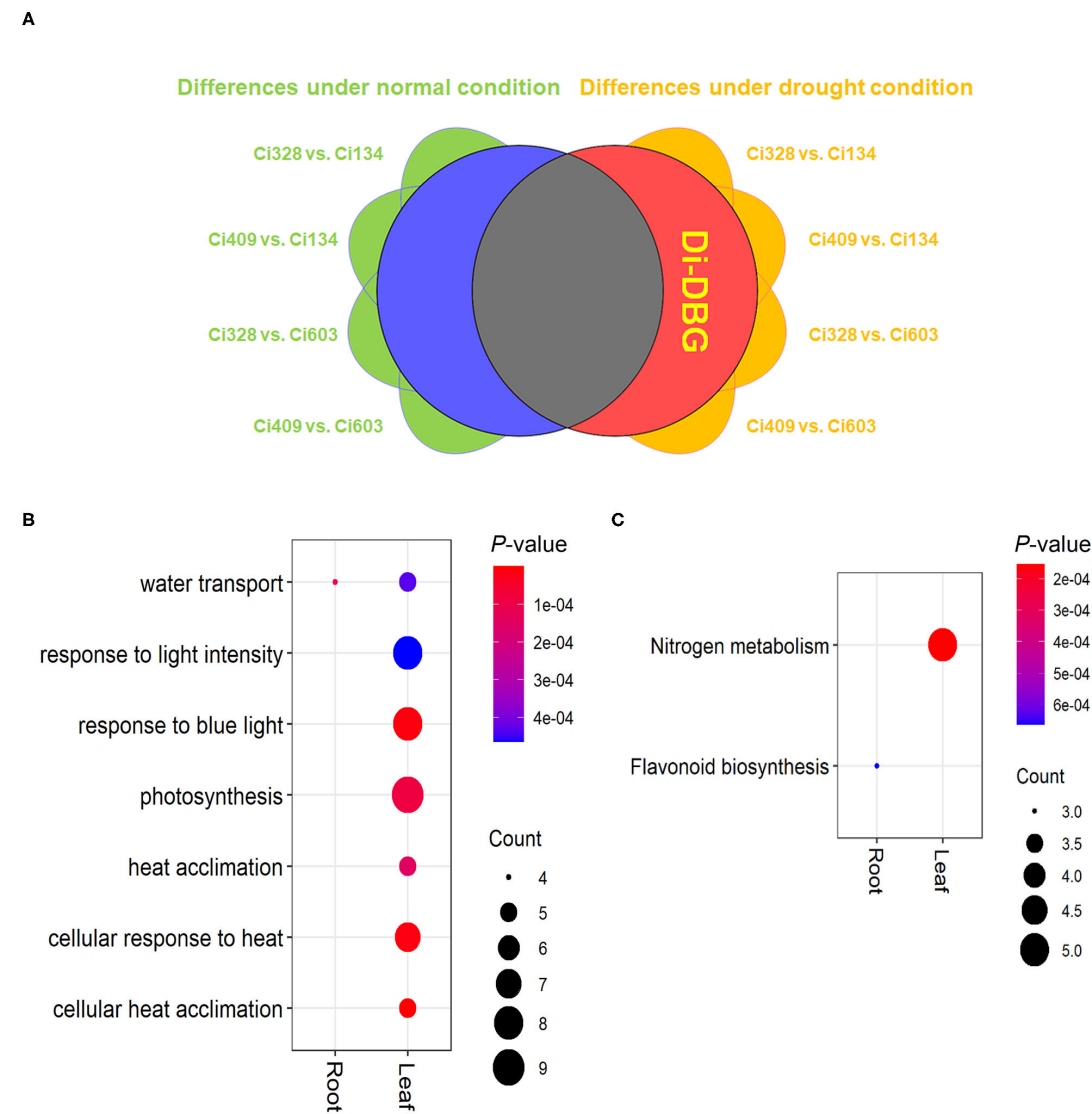
Drought-induced DEGs between genotypes (di-DBG) identified between tolerant and sensitive genotypes. **(A)** The definition and identification of di-DBG. **(B,C)** GO **(B)** and KEGG **(C)** enrichment analysis of di-DBG in leaves and roots.

To achieve a better understanding of the genotypic effect on drought tolerance for all four accessions, we conducted four group comparisons between drought-tolerant and drought-sensitive accessions (Ci328 vs. Ci134, Ci328 vs. Ci603, Ci409 vs. Ci134 and Ci409 vs. Ci603) in all three tissues under both normal and drought conditions, respectively. After eliminating the DEGs that were shared by both normal and drought conditions, a total of 406 (115 in leaves, 79 in stems, and 212 in roots) drought-specific DEGs were screened out, which was defined as drought-induced DEGs between genotypes (di-DBG) (Figure 7A).

The “flavonoid biosynthesis pathway” was enriched in di-DBG identified in roots, and the “nitrogen metabolism pathway” was enriched in di-DBG detected in leaves (Figure 7B). Moreover, the water transport biological process was enriched in di-DBG identified in roots and leaves, photosynthesis, and heat response pathways, which were also enriched in di-DBG detected in leaves (Figure 7C). Furthermore, there were 12 di-DBGs overlapped with DEGs oppositely regulated between tolerant and sensitive genotypes under limited water conditions, which were identified (marked as di-DBG in Supplementary Table S7), such as *HSP70* (*Seita.3G216900*), *HSP23.5* (*Seita.1G326600*), and *SnRK3.16* (*Seita.5G031200*), vital regulators of drought tolerance in plants (Cho et al., 2018; Sewelam et al., 2019; Leonetti et al., 2021).

### Hub Genes Involved in Hormone Homeostasis, Osmotic Adjustment, Post-Transcriptional Modulation, and Photosynthesis Are Essential Regulators of Drought Tolerance

The weighted gene co-expression network analysis (WGCNA) method constructs relationships of expressed genes identified from multi-samples through RNA-sequencing approaches. Totally, 28,401 expressed genes identified in 72 samples were divided into 34 modules (Supplementary Figure S10, Supplementary Table S11). We defined transcripts with connectivity intro-modular (KIM) higher than 0.8 as hub genes (Supplementary Table S11). There was a positive correlation of purple, yellow, midnight blue, and pink modules with the drought treatments of all accessions. There was a negative correlation of orange, red, and green modules with the drought treatment, and a positive correlation of the purple module with soluble sugar content in all accessions was identified (Supplementary Figure S11).

**Figure 8:**
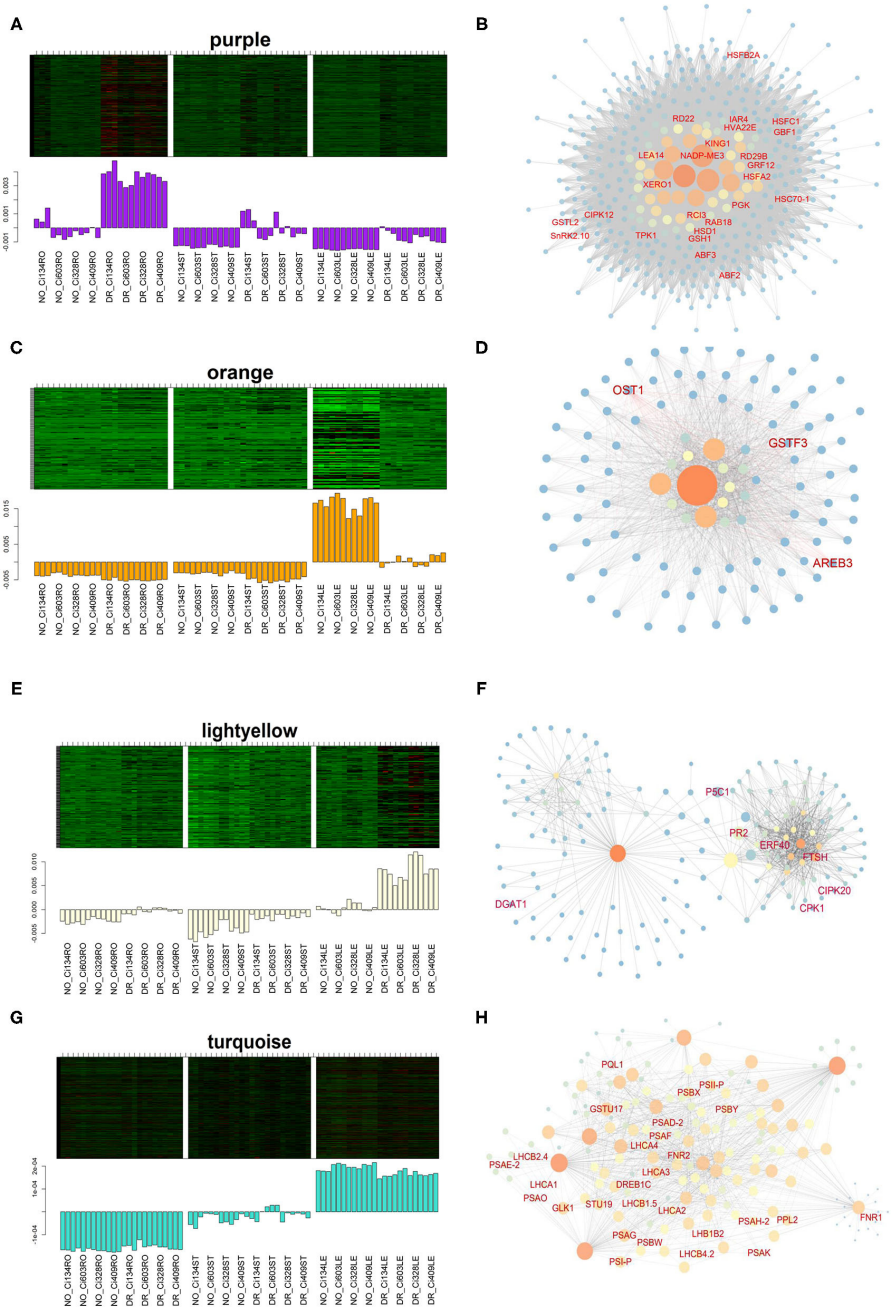
Eigengene expression and the regulatory network of DEGs in drought-correlated modules identified through the WGCNA approach. **(A,B)** Eigengene expression **(A)** and network **(B)** of the purple module; **(C,D)** Eigengene expression **(C)** and the network **(D)** of the purple module; **(E,F)** Eigengene expression **(E)** and the network **(F)** of the light yellow module; **(G,H)** Eigengene expression **(G)** and the network **(H)** of the turquoise module.

Among all drought-responding-related modules, we further analyzed the purple and orange modules. There was a total of 506 genes grouped into the purple module under drought conditions. We identified 17 out of 78 hub genes that are associated with drought tolerance in plant species (Figures 8A,B), such as *CIPK12* (Yasuda et al., 2017), *CBF1* (Wei et al., 2016), *SnRK2.10* (Maszkowska et al., 2019), and *RD22* (Matus et al., 2014), suggesting these gene sets might also be vital to drought tolerance of *S. italica*. The biological processes of “response to water” and “response to abiotic stimulus” were enriched in the purple module (Supplementary Table S12), which implied that the purple module might play an important role in foxtail millet for adjusting drought conditions.

A total of 119 genes grouped into the orange module were downregulated in leaves under drought conditions, and “monobactam biosynthesis” and “lysine biosynthesis” pathways were enriched in this module. Notably, previous studies have shown that many genes grouped into the orange module are key regulators in the stress tolerance of plants (Figures 8C,D). For instance, *AREB3* (*Seita.5G356200*) has been identified as a downstream target of the ABA-signaling pathway and could regulate stomata openness in *Arabidopsis* (Qian et al., 2019). The characterization of *OST1* (*Seita.9G318200*) in rice revealed the functions involved in regulating stomatal closure in response to water-deficient conditions through ABA-signaling modulation (Yoshida et al., 2002). Additionally, *GSTF3* (*Seita.5G171000*) is a glutathione transferase induced by oxidative stress environments in *Arabidopsis* (Lee et al., 2014). Therefore, the orange module might contain vital regulators responsible for drought tolerance of foxtail millet.

Moreover, pink (734 genes, *R* = 0.79, *P* = 3e-16) and yellow (1,966 genes, *R* = 0.77, *P* = 2e-15) modules were also positively correlated with drought treatment in this study. Gene sets grouped into these two modules were mainly involved in RNA processing, transport, and the surveillance process (Supplementary Table S13), implying that post-transcriptional modulation of functional regulators might play important roles in drought tolerance of *S. italica*.

The light yellow module (196 genes, *R* = 0.57, *P* = 2e-7) was also positively correlated with the drought treatment in leaves (Figures 8E,F). The biological processes of “response to heat” and “very-long-chain fatty acid metabolism” were enriched in this module (Supplementary Table S12). The light yellow module contains many vital regulators identified in previous studies, such as *P5C1* (*Seita.5G450800*), which has been confirmed to be associated with the catalysis of the final step of proline biosynthesis and involved in stress-induced proline accumulation in leaves of plants (Giberti et al., 2014). *TINY* (*Seita.5G141600*) is also essential to stress tolerance, as it binds dehydration-responsive and ethylene-responsive *Cis*-elements of viral regulators in *Arabidopsis* (Sun et al., 2008). *CIPK20* (*Seita.5G145900*) encodes a CBL-interacting serine/threonine-protein kinase involved in the ABA-signaling pathway (Wang Z. Y. et al., 2011), and *DGAT1* (*Seita.3G112300*) catalyzes the final step of triacylglycerol assembling, which could contribute to tolerance of freezing in *Arabidopsis* (Arisz et al., 2018).

Interestingly, the turquoise module was negatively correlated with the drought-treated leaves and positively correlated with drought-treated stems and roots (Supplementary Figure S11). The turquoise module was also detected as a downregulated DEG in leaves of all genotypes under drought conditions, and “photosynthesis-related biological processes” were mainly enriched in this module. This result from the turquoise module yielded more than 20 DEGs functions as components of PSI, PSII, and the light-harvesting complex (Figures 8G,H), which is consistent with decreased photosynthetic rates observed in all four accessions under drought conditions (Figure 1E). Moreover, most photosynthesis-related genes have also been downregulated in the leaves of foxtail millet under drought conditions at the jointing stage (Supplementary Figure S12), suggesting that photosynthesis-related traits change dramatically under water-limited conditions in foxtail millet.

### Plant Hormone Pathways Were Conserved During Multiple Growth Stages in Foxtail Millet

Given that previous study about drought tolerance of *S. italica* mainly focused on the aboveground part of plant individuals, we have compared DEGs detected in leaves with the previously reported dataset (including 3 seedling stages and 1 germination stage) (Qi et al., 2013; Tang et al., 2017; Qin et al., 2020; Yu et al., 2020). We found 10 shared genes in response to osmotic stress during germination, seedling, and the jointing stage in foxtail millet, including *HAB1* (*Seita.3G164700*, Lim and Lee, 2020), *AREB1* (*Seita.4G082000*, Yoshida et al., 2010), *GBF3* (*Seita.5G251200*, Ramegowda et al., 2017), *AHG1* (*Seita.6G005300*, Nishimura et al., 2007), and *HAI2* (*Seita.9G460200*, Lim et al., 2012) that participate in the ABA pathway in plants, and *LEA3-4* (*Seita.5G287800*) induced by multi abiotic stresses (Zhao et al., 2011) and 4 more genes with unknown functions. A total of 193 jointing stage-specific DEGs were identified, including 14 DEGs involved in plant hormone regulation, 14 DEGs in response to abiotic stress, and 5 DEGs participating in photosynthesis, implying different mechanisms of drought tolerance between lateral and early growth stages in foxtail millet (Supplementary Table S14). However, due to the different genotypes tested in previous studies, and genotype-based differences could not be eliminated in the comparison analysis, further researches are still needed in the future for clarifying the unique drought-tolerant mechanisms in foxtail millet among different developmental stages.

### Verification of RNA-Seq Results Through the qRT-PCR Approach

The randomly selected DEGs in response to drought treatments from different tissues were used for qRT-PCR assays to verify the reliability of RNA-seq data. We randomly selected nine DEGs from leaves, four DEGs from stems, and six DEGs from roots, including 1 DEG (*Seita.9G095900)* with the opposite pattern in response to drought for further analysis. The results of qRT-PCR were highly consistent with RNA-seq data with *R* = 0.787, indicating that the RNA-seq results obtained in this trial are reliable (Supplementary Figure S12, Supplementary Table S15).

## Conclusion

*Setaria italica* is known as one of the most drought-tolerant cereal species. In this trial, we selected two drought-tolerant and two drought-sensitive accessions of *S. italica* for the exploration of drought-tolerant regulators in this crop species. Under drought stress conditions, the drought-sensitive genotypes heavily lost grain yield and biomass, while the drought-tolerant genotypes showed more stable growth and yield. RNA-seq analysis of three tissues under drought conditions showed most DEGs were identified in a tissue-specific manner, and the number of DEGs was higher in roots, followed by leaves and stems. Functional enrichment analysis revealed that genes related to photosynthesis in leaves, elongation of the stem, and the growth of roots were repressed by drought stress. We only identified a few DEGs overlapped in the three tissues, which implies that different tissues take different actions to achieve similar goals of drought tolerance in foxtail millet. Comparisons of genes expression profiling between tolerant and sensitive accessions indicated 20 drought-induced regulators contributing to genotypic variations of drought tolerance. WGCNA identified 34 modules and 1,343 hub genes playing critical roles in the drought tolerance of *S. italica*.

## Data Availability Statement

The datasets presented in this study can be found in online repositories. The names of the repository and accession number can be found at: https://www.ebi.ac.uk/ena, PRJEB43702.

## Author Contributions

XD and GJ supervised the experiments. RZ, HZ, YL, EG, GF, ST, WG, and LZ performed the experiments. RZ and GJ analyzed the data. RZ, GJ, and XD wrote the manuscript. All authors contributed to the article and approved the submitted version.

## Funding

This study was supported by the National Key R&D Program of China (2018YFD1000701/2018YFD1000700), National Natural Science Foundation of China (31871630), China Agriculture Research System of MOF and MARA (CARS07-13.5-A04), and the Agricultural Science and Technology Innovation Program of the Chinese Academy of Agricultural Sciences.

## Conflict of Interest

The authors declare that the research was conducted in the absence of any commercial or financial relationships that could be construed as a potential conflict of interest.

## Publisher's Note

All claims expressed in this article are solely those of the authors and do not necessarily represent those of their affiliated organizations, or those of the publisher, the editors and the reviewers. Any product that may be evaluated in this article, or claim that may be made by its manufacturer, is not guaranteed or endorsed by the publisher.

## Supplementary Material

Supplementary Figure S1 The pipeline of datasets analysis. After sample harvest and RNA-seq, expression data were generated for further analysis. DEGs were screened through two approaches: drought condition vs. normal condition and tolerant genotypes vs. sensitive genotypes. The former strategy revealed differences in drought response between genotypes and tissues. Additionally, the latter strategy revealed differences between genotypes induced by drought. The co-expression network (gene modules) was generated by WGCNA. Hub genes of drought-correlated modules were then identified. Functional enrichment analysis was conducted for dissection of drought-tolerant mechanisms in foxtail millet.

Supplementary Figure S2 Drought treatment and sampling of foxtail millet tissues for RNA-seq analysis. **(A)** Strategy for water management. Drought treatment was conducted after 45 days of germination; **(B)** Sampled tissues for further analysis. Two upper fully expanded leaves, elongated stems, and all roots were sampled for RNA-seq analysis.

Supplementary Figure S3 Functional enrichment of DEGs identified in the roots. KEGG **(A)** and GO **(B)** enrichment analysis for DEGs detected in all genotypes. Each column represents different gene sets; circle size represents the gene ratio of DEGs in each category to all annotated genes; circle color represents the *P*-value; the number located to each circle represents the amount of DEGs.

Supplementary Figure S4 Functional enrichment of DEGs identified in the stems. KEGG **(A)** and GO **(B)** enrichment analysis for DEGs detected in all genotypes. Each column represents different gene sets; circle size represents the gene ratio of DEGs in each category to all annotated genes; circle color represents the *P*-value; the number located to each circle represents the amount of DEGs.

Supplementary Figure S5 Functional enrichment of DEGs identified in the leaves. KEGG **(A)** and GO **(B)** enrichment analysis for DEGs detected in all genotypes. Each column represents different gene sets; circle size represents the gene ratio of DEGs in each category to all annotated genes; circle color represents *P*-value; the number located to each circle represents the amount of DEGs.

Supplementary Figure S6 Comparisons of upregulated DEGs identified in all three tissues sampled from all four accessions. Up four Venn diagrams showed DEGs induced by drought among genotypes of **(A)** Ci134, **(B)** Ci603, **(C)** Ci328, and **(D)** Ci409, and below three ones showed DEGs induced by drought in tissues of **(E)** root, **(F)** stem, and **(G)** leaf. Numbers in parentheses refer to total DEGs detected in each circle.

Supplementary Figure S7 Comparisons of downregulated DEGs identified in all three tissues sampled from all four accessions. Up four Venn diagrams showed DEGs repressed by drought among genotypes of **(A)** Ci134, **(B)** Ci603, **(C)** Ci328, and **(D)** Ci409, and below three ones showed DEGs repressed by drought in tissues of **(E)** root, **(F)** stem, and **(G)** leaf. Numbers in parentheses refer to total DEGs detected in each circle.

Supplementary Figure S8 Functional enrichment of genotype-specific DEGs. **(A)** Relationships among sensitive-genotype-specific DEGs (Se-specific, blue color), tolerant-genotype-specific DEGs (To-specific, red color), and all genotype-shared DEGs (all-shared, yellow color); **(B,C)** GO **(B)** and KEGG **(C)** enrichment analysis of DEGs detected in both tolerant and sensitive genotypes. “To” represents drought-tolerant genotypes, including Ci328 and Ci409; “Se” represents drought-sensitive genotypes, including Ci134 and Ci603. Each column represents different gene sets, circle size represents the gene ratio of DEGs in each category to all annotated genes, circle color represents the *P*-value, and the number located to each circle represents the amount of DEGs.

Supplementary Figure S9 Heat-map of genotype-shared DETFs. The heat map of 200 DETFs detected in all genotypes. Red color refers to upregulation, while blue color refers to downregulation.

Supplementary Figure S10 Construction of the weighted gene co-expression network with all expressed genes identified among all samples. **(A)** The clustering tree of 72 samples used FPKM values of all expressed genes; blue and red below the clustered tree represents control and drought treatments in this trial, respectively; **(B,C)** a soft threshold determined through scale independence **(B)** and mean connectivity **(C)**; **(D)** the clustering tree of all expressed genes; each color represents a module. NO refers to normal, and DR refers to drought conditions. RO refers to the root, ST refers to the stem, and LE refers to the leaf.

Supplementary Figure S11 The heatmap of module-trait relationships for all modules. Red and blue represent positive or negative correlations of modules and traits, respectively. The upper number in each cell is the *R* of correlation; the lower number between parentheses is the *P*-value. RLWC: relative leaf water content; LWP: leaf water potential; SSC: soluble sugar content.

Supplementary Figure S12 Validations of DEGs by the qRT-PCR approach. Positive numbers and red color refer to upregulation; negative numbers and blue color refer to downregulation. Log_2_FC featured RNA-seq and qRT-PCR featured by –ΔΔCT, calculated from the relative expression of four individual replications.

Supplementary Table S1 Quality evaluation of RNA-seq raw data.

Supplementary Table S2 FPKMs for all annotated genes identified in this trial.

Supplementary Table S3 DEGs identified in all samples between normal and drought conditions.

Supplementary Table S4 A list of KEGG categories of DEGs identified in this trial.

Supplementary Table S5 A list of GO categories of DEGs detected in this study.

Supplementary Table S6 A list of GO and KEGG categories of genotype-specific DEGs.

Supplementary Table S7 Oppositely regulated DEGs identified between drought-tolerant and sensitive genotypes.

Supplementary Table S8 DETFs detected in both drought-tolerant and sensitive genotypes.

Supplementary Table S9 K-means clustering in this trial.

Supplementary Table S10 A list of GO and KEGG categories of k-means clusters.

Supplementary Table S11 Results of WGCNA and KIM of all genes in each module.

Supplementary Table S12 A list of GO categories of each module.

Supplementary Table S13 A list of KEGG categories of each module.

Supplementary Table S14 Comparisons of drought-responsive genes in leaves between different development stages of *Setaria italica*.

Supplementary Table S15 Validation of DEGs by qRT-PCR in this study.
