# Supplementary figures and images for: Response of Multiple Tissues to Drought Revealed by a Weighted Gene Co-Expression Network Analysis in Foxtail Millet [Setaria italica (L.) P. Beauv.]
Source: Front Plant Sci. 2022 Jan 12;12:746166. doi: 10.3389/fpls.2021.746166 (PMC8790073; doi:10.3389/fpls.2021.746166)

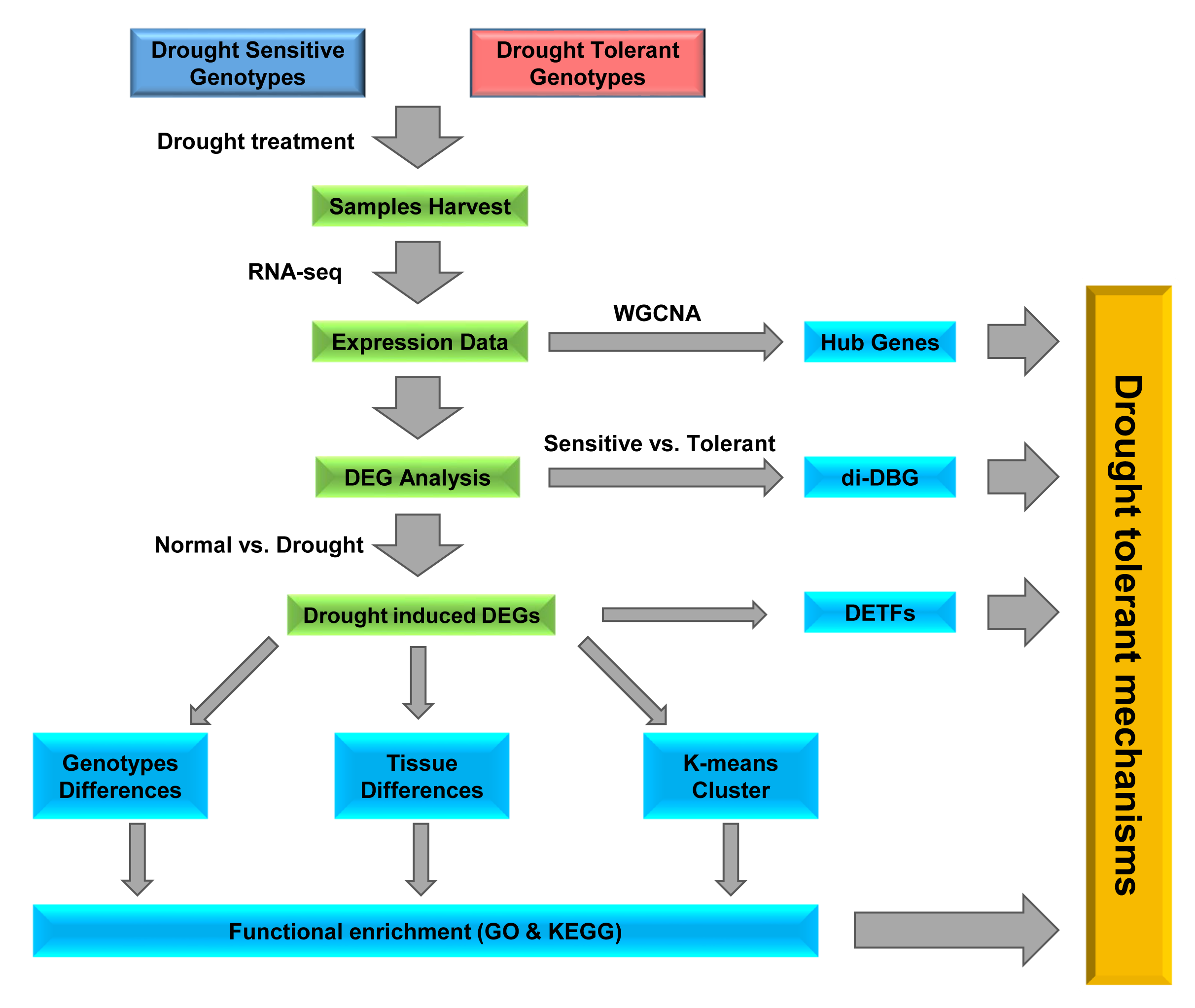

Supplement: Supplementary Figure S1 — The pipeline of datasets analysis. After sample harvest and RNA-seq, expression data were generated for further analysis. DEGs were screened through two approaches: drought condition vs. normal condition and tolerant genotypes vs. sensitive genotypes. The former strategy revealed differences in drought response between genotypes and tissues. Additionally, the latter strategy revealed differences between genotypes induced by drought. The co-expression network (gene modules) was generated by WGCNA. Hub genes of drought-correlated modules were then identified. Functional enrichment analysis was conducted for dissection of drought-tolerant mechanisms in foxtail millet. [file Image_1.TIF]

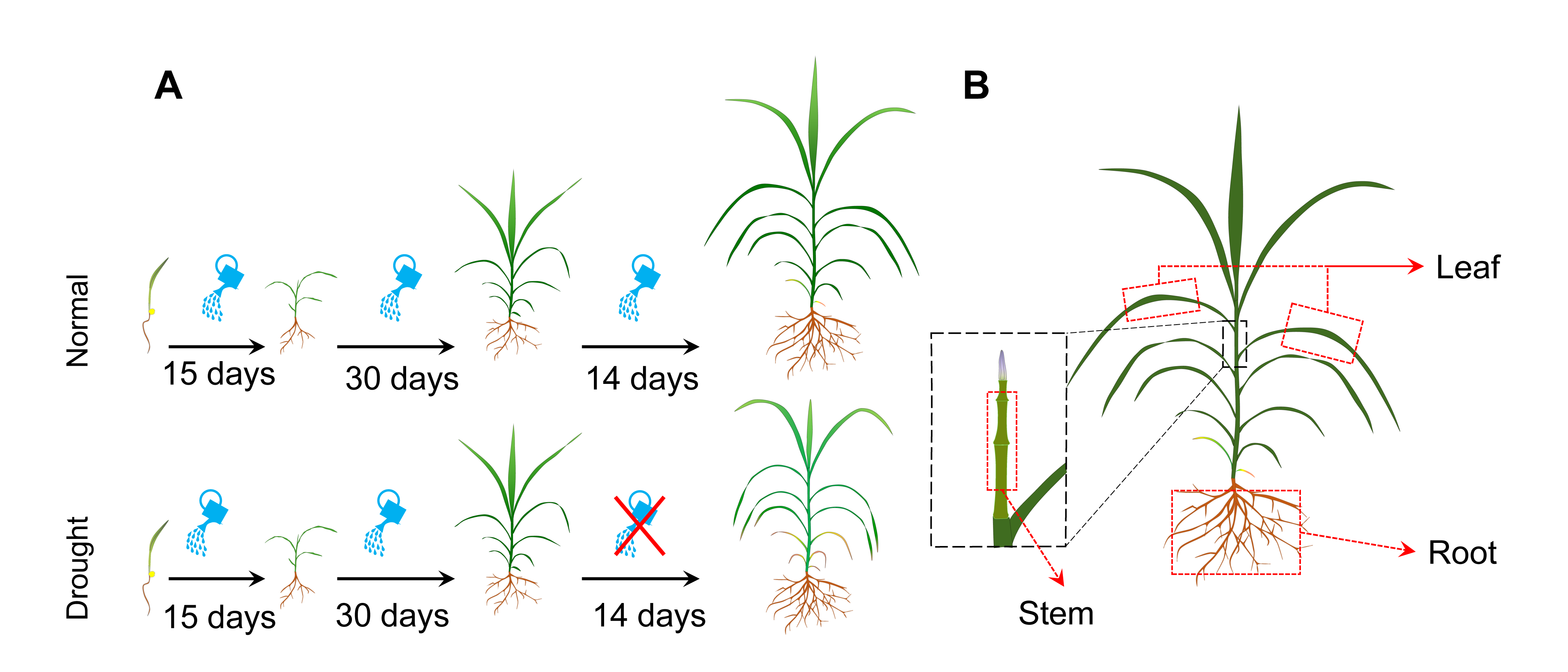

Supplement: Supplementary Figure S2 — Drought treatment and sampling of foxtail millet tissues for RNA-seq analysis. (A) Strategy for water management. Drought treatment was conducted after 45 days of germination; (B) Sampled tissues for further analysis. Two upper fully expanded leaves, elongated stems, and all roots were sampled for RNA-seq analysis. [file Image_2.TIF]

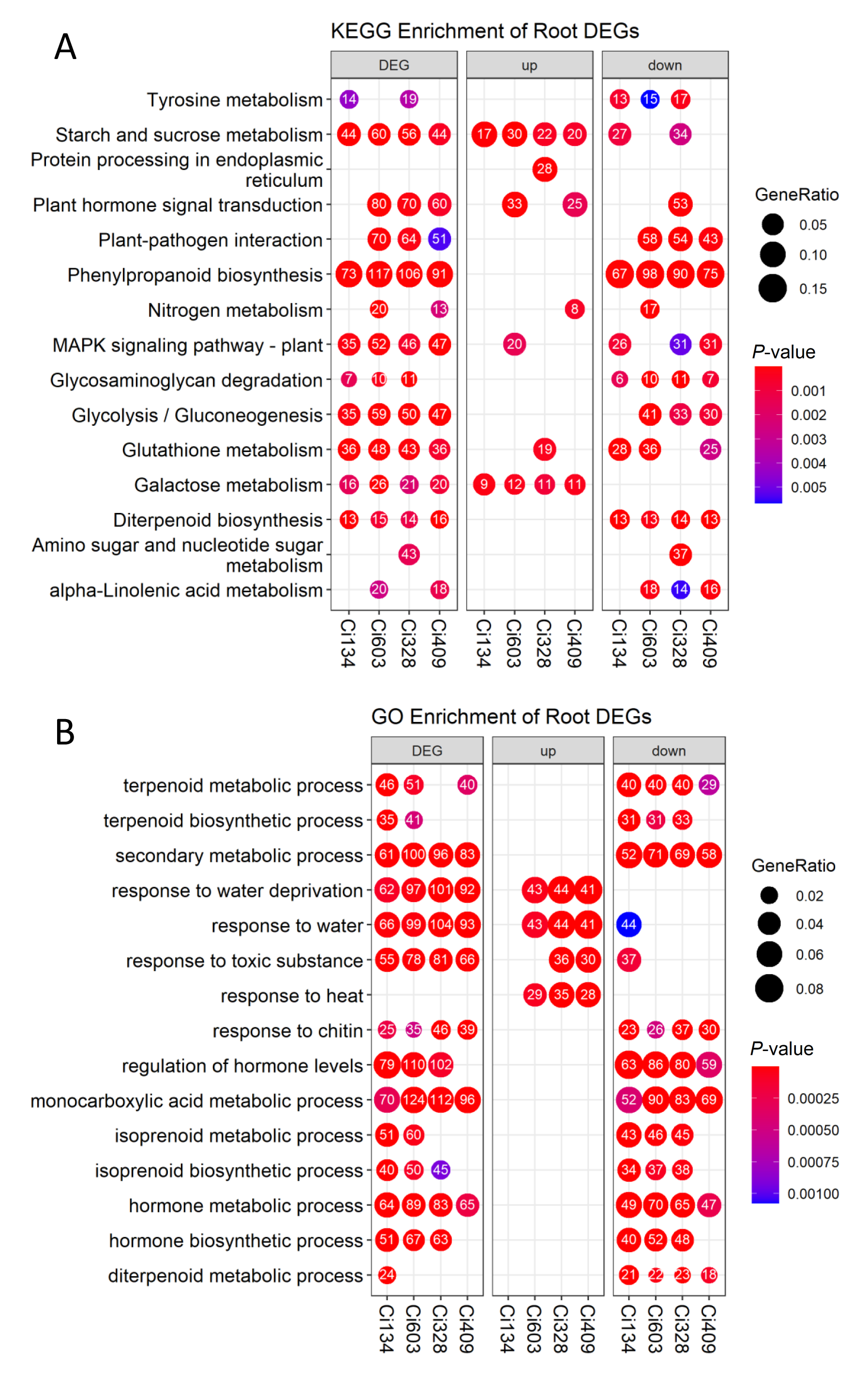

Supplement: Supplementary Figure S3 — Functional enrichment of DEGs identified in the roots. KEGG (A) and GO (B) enrichment analysis for DEGs detected in all genotypes. Each column represents different gene sets; circle size represents the gene ratio of DEGs in each category to all annotated genes; circle color represents the P-value; the number located to each circle represents the amount of DEGs. [file Image_3.TIF]

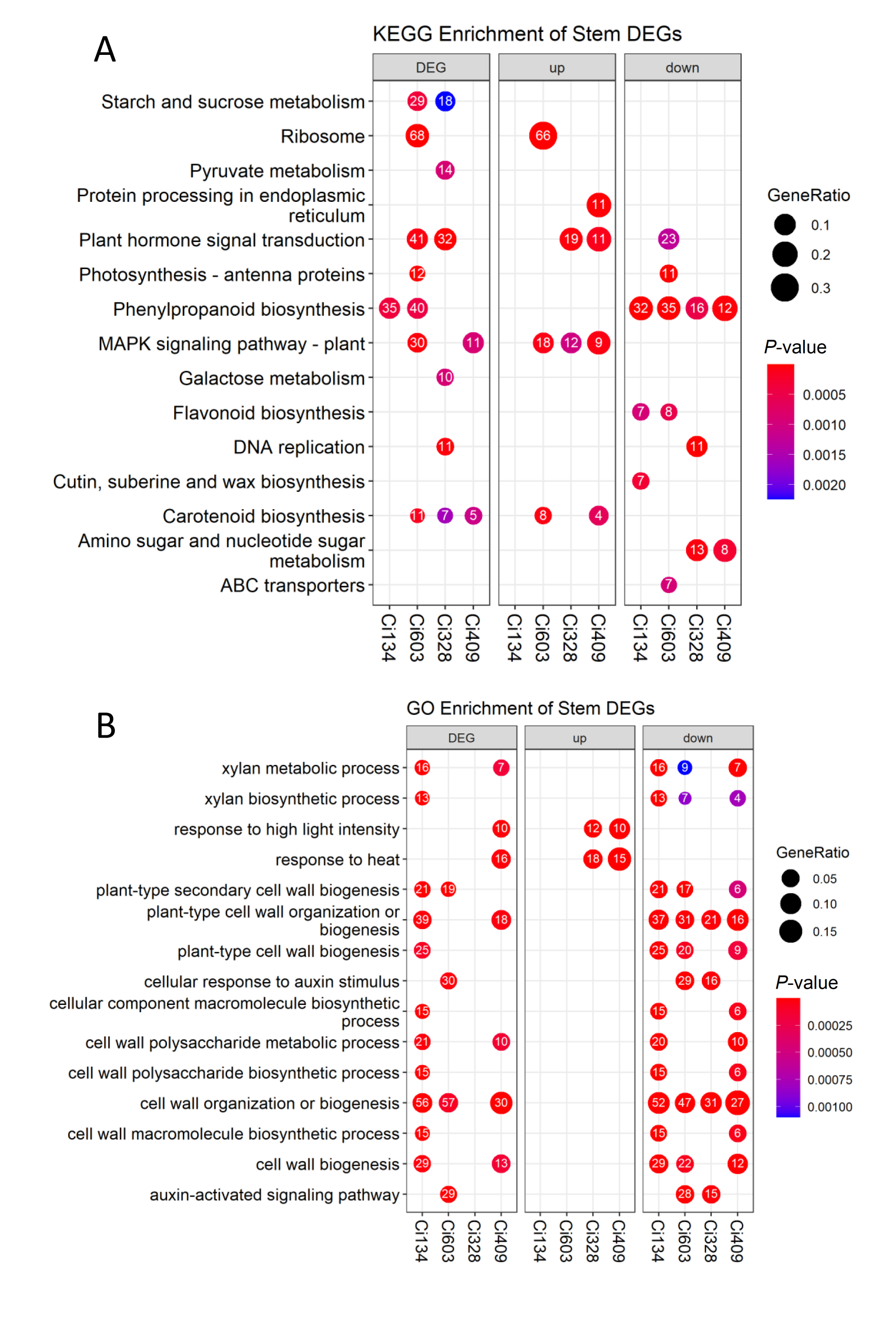

Supplement: Supplementary Figure S4 — Functional enrichment of DEGs identified in the stems. KEGG (A) and GO (B) enrichment analysis for DEGs detected in all genotypes. Each column represents different gene sets; circle size represents the gene ratio of DEGs in each category to all annotated genes; circle color represents the P-value; the number located to each circle represents the amount of DEGs. [file Image_4.TIF]

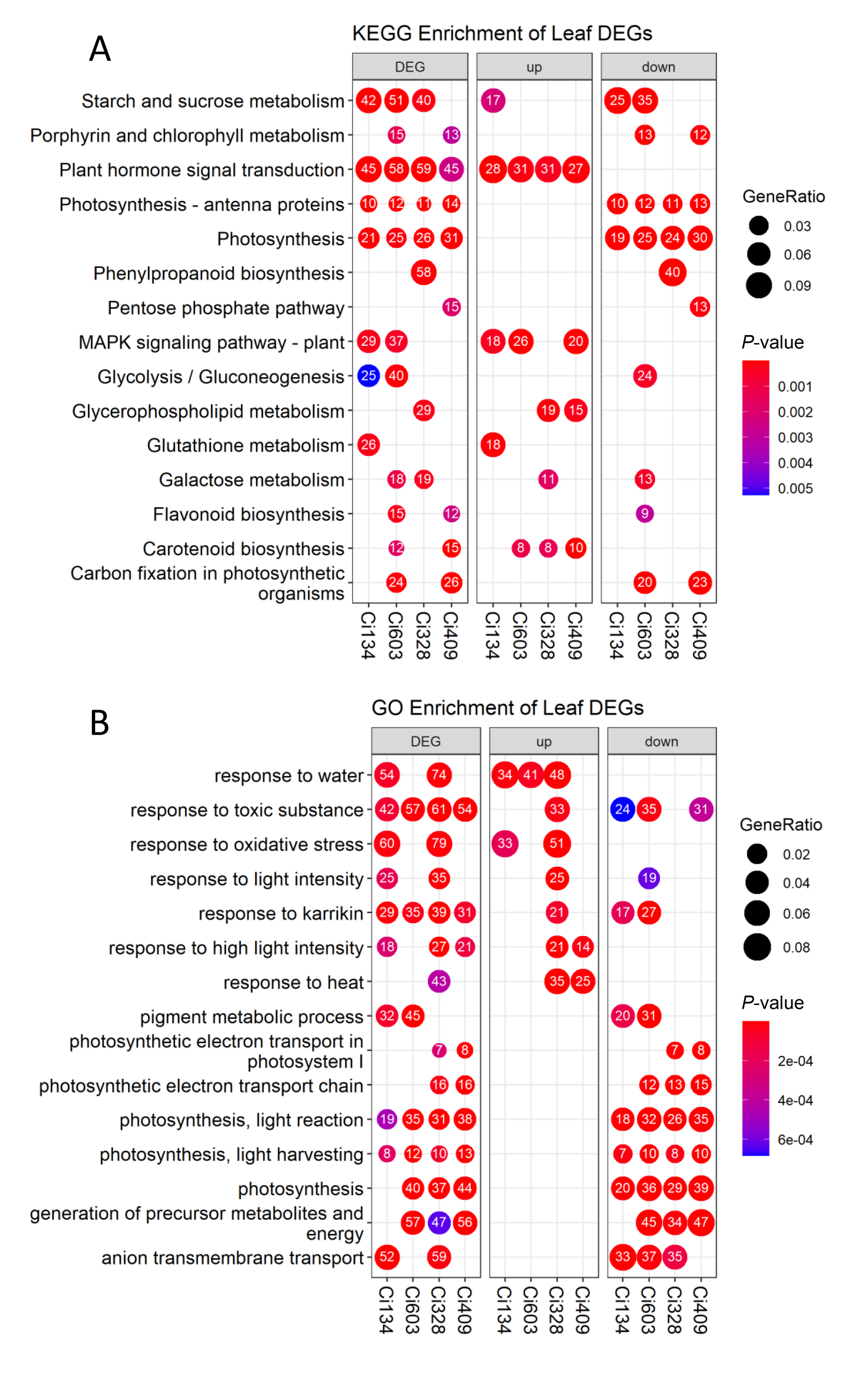

Supplement: Supplementary Figure S5 — Functional enrichment of DEGs identified in the leaves. KEGG (A) and GO (B) enrichment analysis for DEGs detected in all genotypes. Each column represents different gene sets; circle size represents the gene ratio of DEGs in each category to all annotated genes; circle color represents P-value; the number located to each circle represents the amount of DEGs. [file Image_5.TIF]

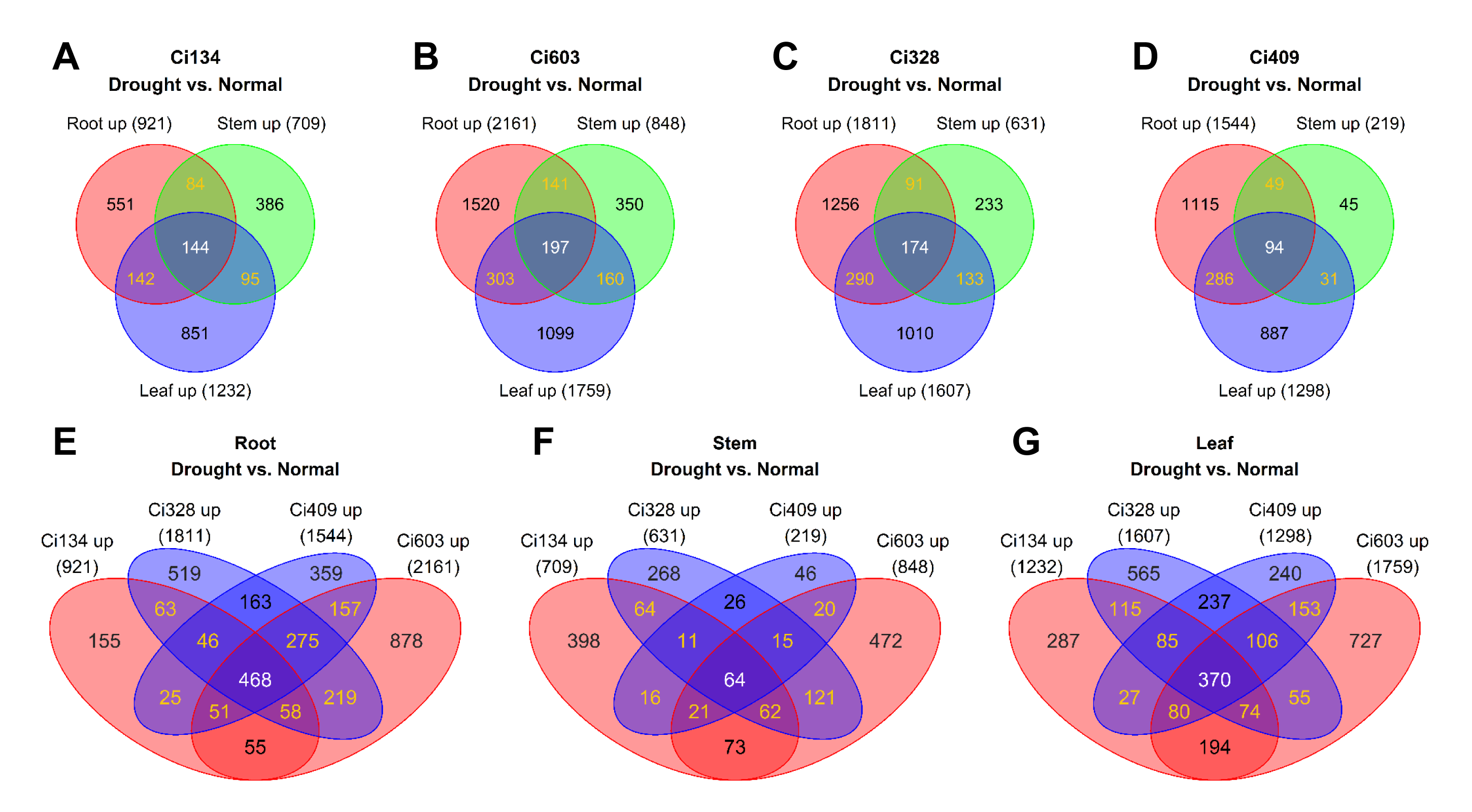

Supplement: Supplementary Figure S6 — Comparisons of upregulated DEGs identified in all three tissues sampled from all four accessions. Up four Venn diagrams showed DEGs induced by drought among genotypes of (A) Ci134, (B) Ci603, (C) Ci328, and (D) Ci409, and below three ones showed DEGs induced by drought in tissues of (E) root, (F) stem, and (G) leaf. Numbers in parentheses refer to total DEGs detected in each circle. [file Image_6.TIF]

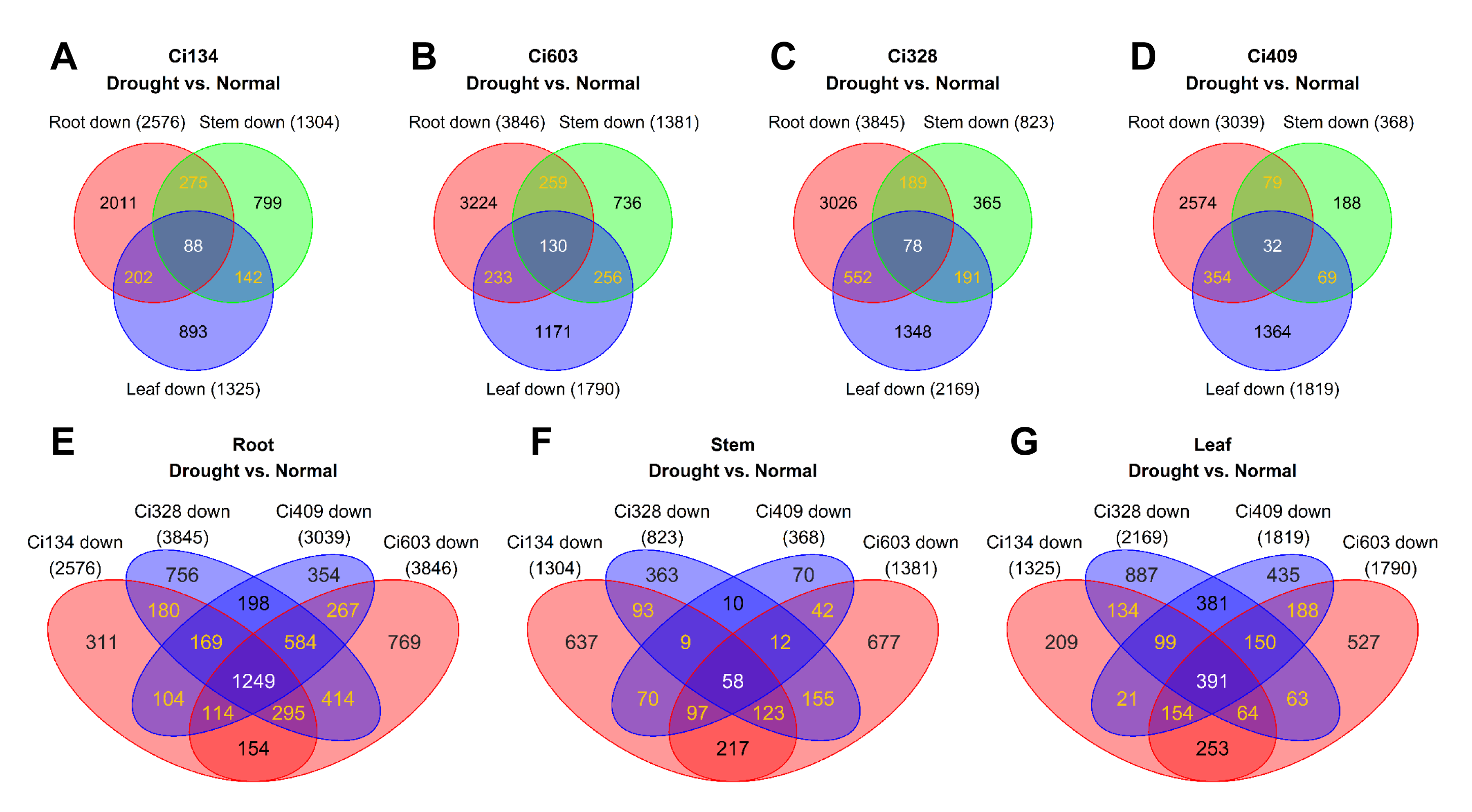

Supplement: Supplementary Figure S7 — Comparisons of downregulated DEGs identified in all three tissues sampled from all four accessions. Up four Venn diagrams showed DEGs repressed by drought among genotypes of (A) Ci134, (B) Ci603, (C) Ci328, and (D) Ci409, and below three ones showed DEGs repressed by drought in tissues of (E) root, (F) stem, and (G) leaf. Numbers in parentheses refer to total DEGs detected in each circle. [file Image_7.TIF]

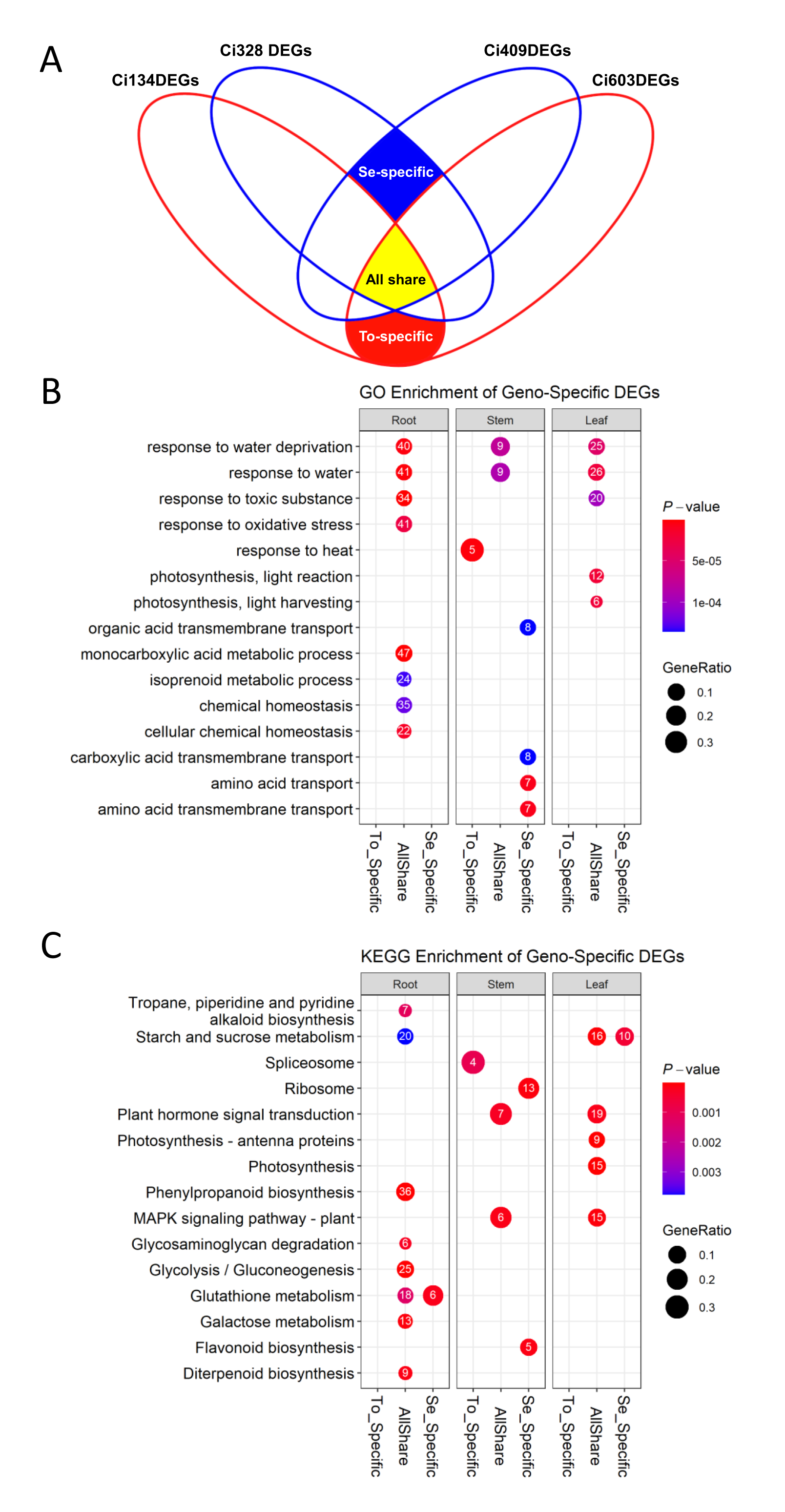

Supplement: Supplementary Figure S8 — Functional enrichment of genotype-specific DEGs. (A) Relationships among sensitive-genotype-specific DEGs (Se-specific, blue color), tolerant-genotype-specific DEGs (To-specific, red color), and all genotype-shared DEGs (all-shared, yellow color); (B,C) GO (B) and KEGG (C) enrichment analysis of DEGs detected in both tolerant and sensitive genotypes. “To” represents drought-tolerant genotypes, including Ci328 and Ci409; “Se” represents drought-sensitive genotypes, including Ci134 and Ci603. Each column represents different gene sets, circle size represents the gene ratio of DEGs in each category to all annotated genes, circle color represents the P-value, and the number located to each circle represents the amount of DEGs. [file Image_8.TIF]

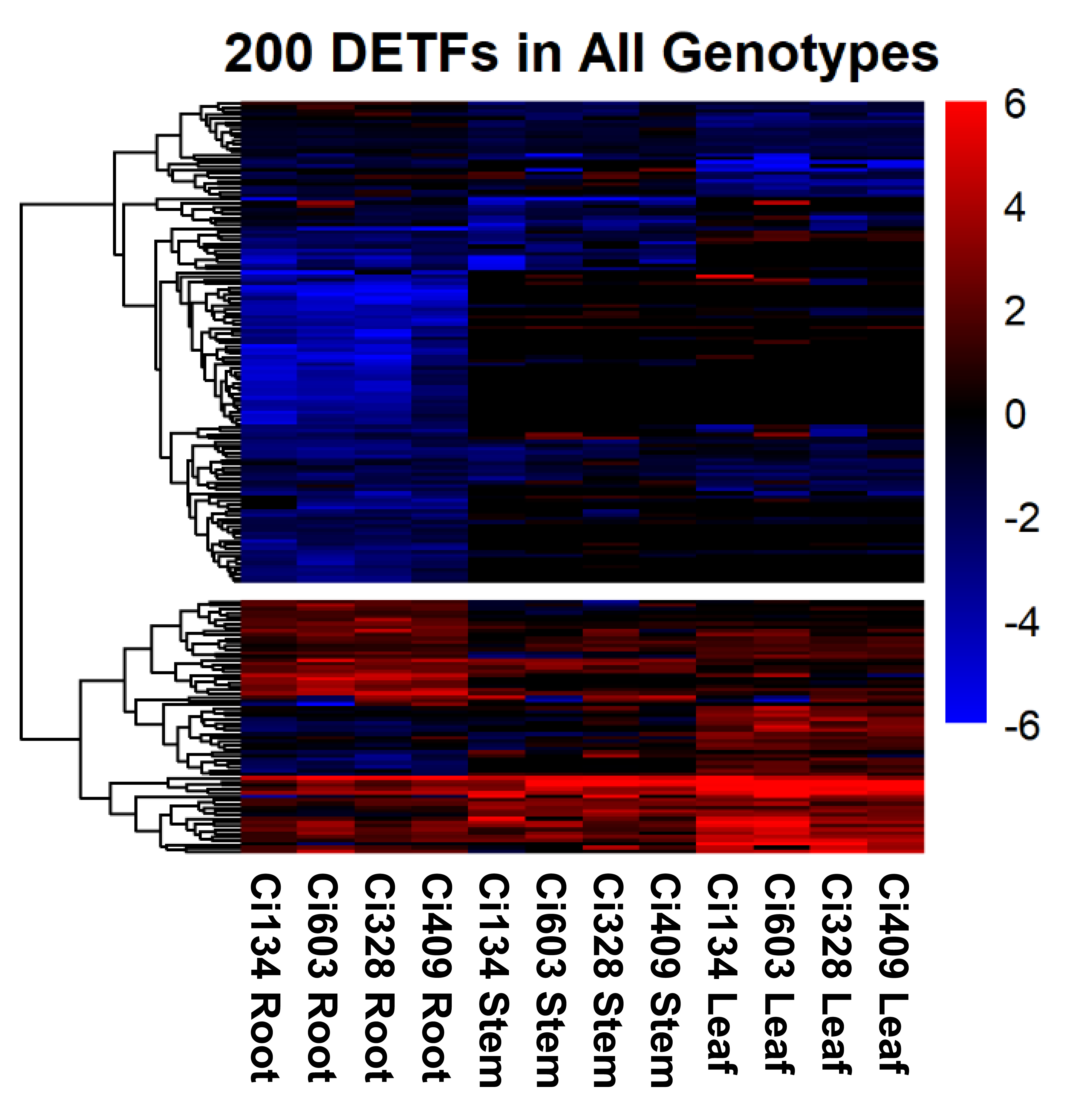

Supplement: Supplementary Figure S9 — Heat-map of genotype-shared DETFs. The heat map of 200 DETFs detected in all genotypes. Red color refers to upregulation, while blue color refers to downregulation. [file Image_9.TIF]

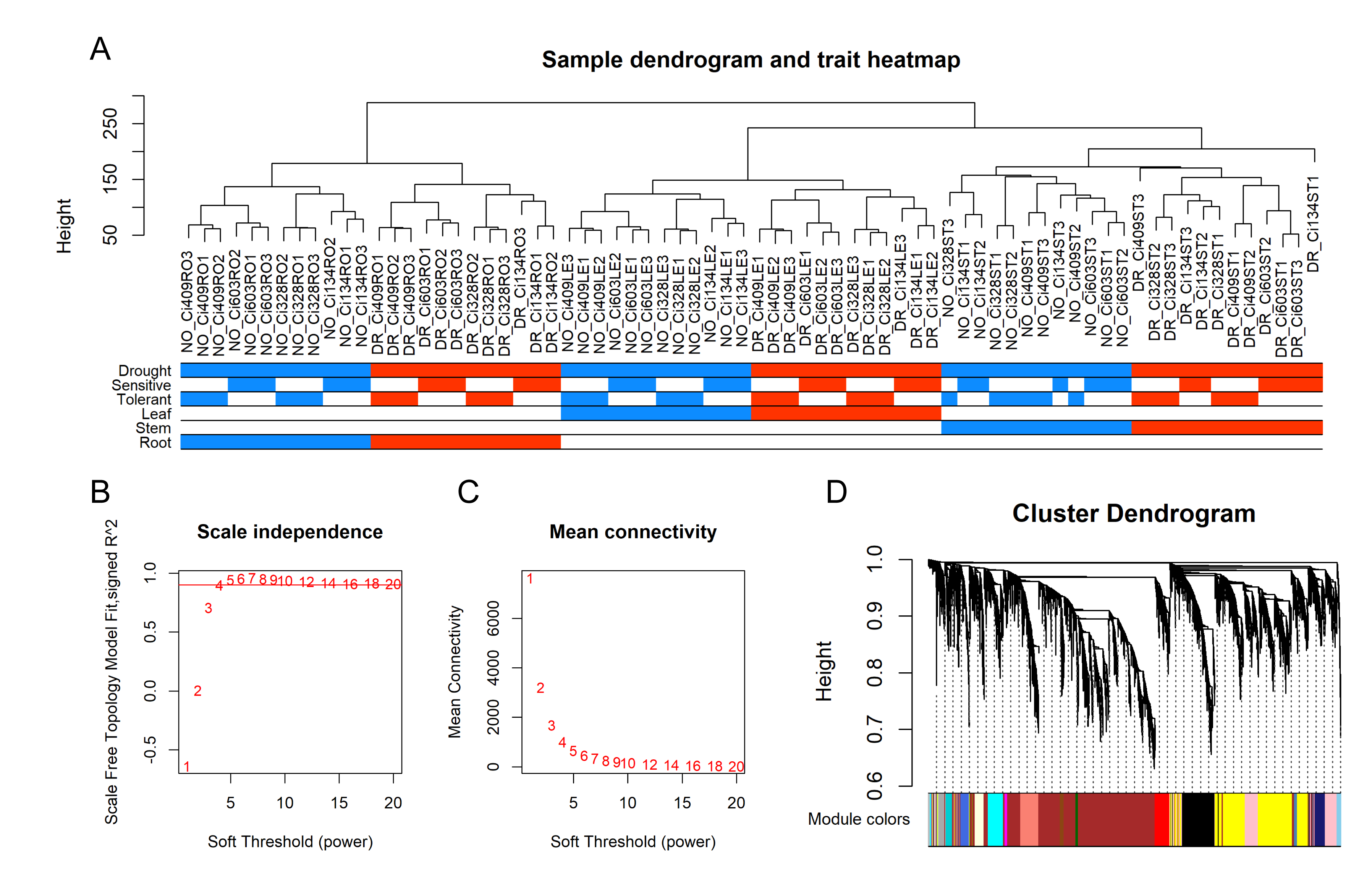

Supplement: Supplementary Figure S10 — Construction of the weighted gene co-expression network with all expressed genes identified among all samples. (A) The clustering tree of 72 samples used FPKM values of all expressed genes; blue and red below the clustered tree represents control and drought treatments in this trial, respectively; (B,C) a soft threshold determined through scale independence (B) and mean connectivity (C); (D) the clustering tree of all expressed genes; each color represents a module. NO refers to normal, and DR refers to drought conditions. RO refers to the root, ST refers to the stem, and LE refers to the leaf. [file Image_10.TIF]

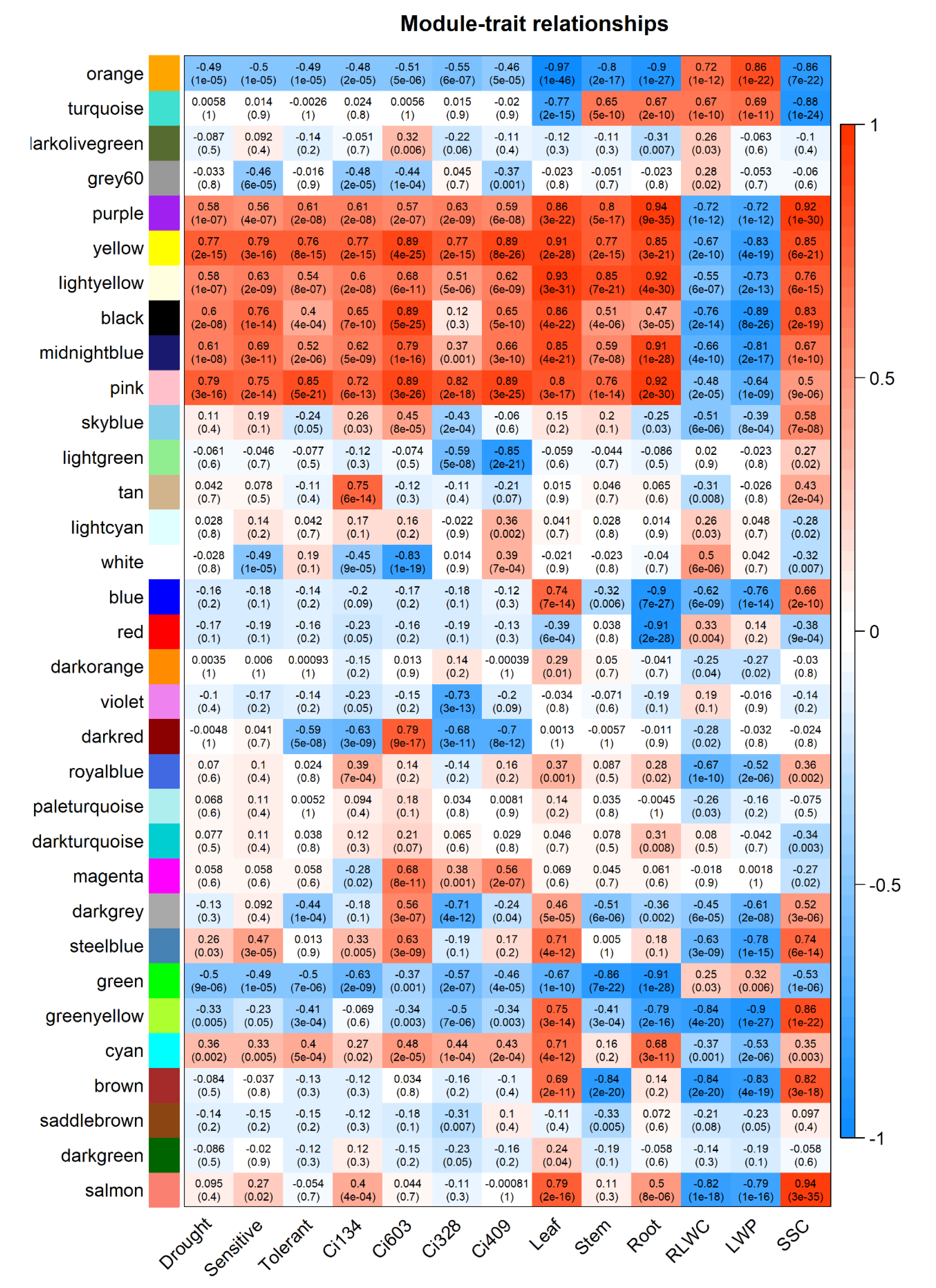

Supplement: Supplementary Figure S11 — The heatmap of module-trait relationships for all modules. Red and blue represent positive or negative correlations of modules and traits, respectively. The upper number in each cell is the R of correlation; the lower number between parentheses is the P-value. RLWC: relative leaf water content; LWP: leaf water potential; SSC: soluble sugar content. [file Image_11.TIF]

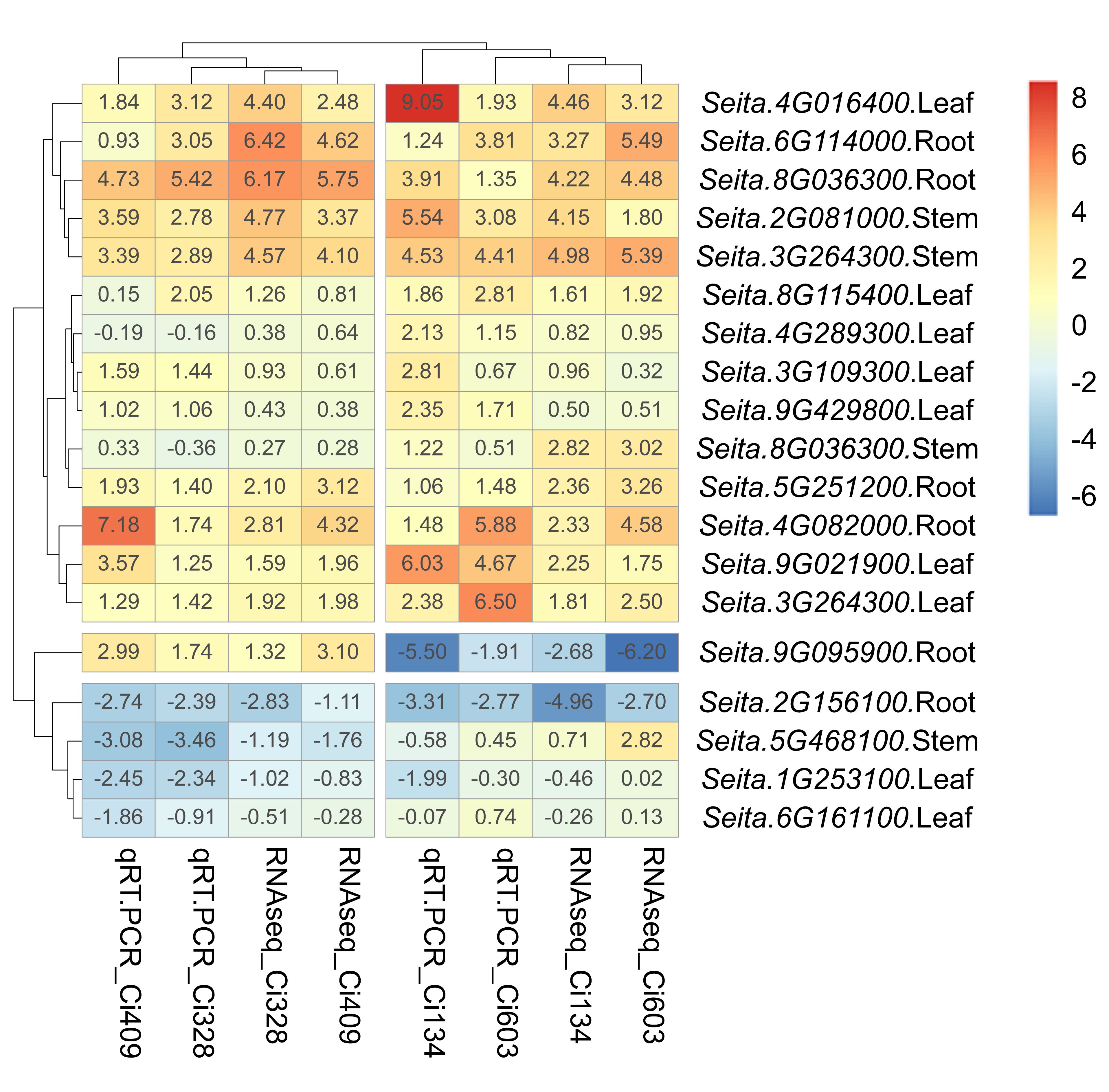

Supplement: Supplementary Figure S12 — Validations of DEGs by the qRT-PCR approach. Positive numbers and red color refer to upregulation; negative numbers and blue color refer to downregulation. Log2FC featured RNA-seq and qRT-PCR featured by –ΔΔCT, calculated from the relative expression of four individual replications. [file Image_12.TIF]
